# Targeting Kruppel-like Factor 9 in Excitatory Neurons Protects against Chronic Stress-Induced Impairments in Dendritic Spines and Fear Responses

**DOI:** 10.1016/j.celrep.2018.05.040

**Published:** 2018-06-12

**Authors:** Antoine Besnard, Tomer Langberg, Sally Levinson, Duong Chu, Cinzia Vicidomini, Kimberly N. Scobie, Andrew J. Dwork, Victoria Arango, Gorazd B. Rosoklija, J. John Mann, René Hen, E. David Leonardo, Maura Boldrini, Amar Sahay

**Affiliations:** 1 Center for Regenerative Medicine, Massachusetts General Hospital, Boston, MA 02114, USA; 2 Harvard Stem Cell Institute, Cambridge, MA 02138, USA; 3 Department of Psychiatry, Massachusetts General Hospital, Harvard Medical School, Boston, MA 02114 USA; 4 BROAD Institute of Harvard and MIT, Cambridge, MA 02142, USA; 5 Department of Psychiatry, Columbia University Medical Center, New York, NY 10032, USA; 6 Department of Pathology and Cell Biology, Columbia University Medical Center, New York, NY 10032, USA; 7 Department of Neuroscience, Columbia University Medical Center, New York, NY 10032, USA; 8 Department of Pharmacology, Columbia University Medical Center, New York, NY 10032, USA; 9 Divisions of Molecular Imaging and Neuropathology, New York State Psychiatric Institute, New York, NY 10032, USA; 10 Division of Integrative Neuroscience, New York State Psychiatric Institute, New York, NY 10032, USA; 11 Macedonian Academy of Sciences & Arts, Skopje 1000, Republic of Macedonia; 12 Lead Contact

## Abstract

Stress exposure is associated with the pathogenesis of psychiatric disorders, including post-traumatic stress disorder (PTSD) and major depressive disorder (MDD). Here, we show in rodents that chronic stress exposure rapidly and transiently elevates hippocampal expression of Kruppel-like factor 9 (*Klf9*). Inducible genetic silencing of *Klf9* expression in excitatory forebrain neurons in adulthood prior to, but not after, onset of stressor prevented chronic restraint stress (CRS)-induced potentiation of contextual fear acquisition in female mice and chronic corticosterone (CORT) exposure-induced fear generalization in male mice. *Klf9* silencing prevented chronic CORT and CRS induced enlargement of dendritic spines in the ventral hippocampus of male and female mice, respectively. *KLF9* mRNA density was increased in the anterior dentate gyrus of women, but not men, with more severe recent stressful life events and increased mortality. Thus, *Klf9* functions as a stress-responsive transcription factor that mediates circuit and behavioral resilience in a sex-specific manner.

## In Brief

Besnard et al. show that chronic stress induces a transient elevation in hippocampal *Klf9* expression in mice and that KLF9 expression is upregulated in hippocampus of women with MDD. Genetic silencing of *Klf9* expression prevents chronic stress-induced enlargements of dendritic spines and maladaptive fear responses in male and female mice.

## Graphical Abstract

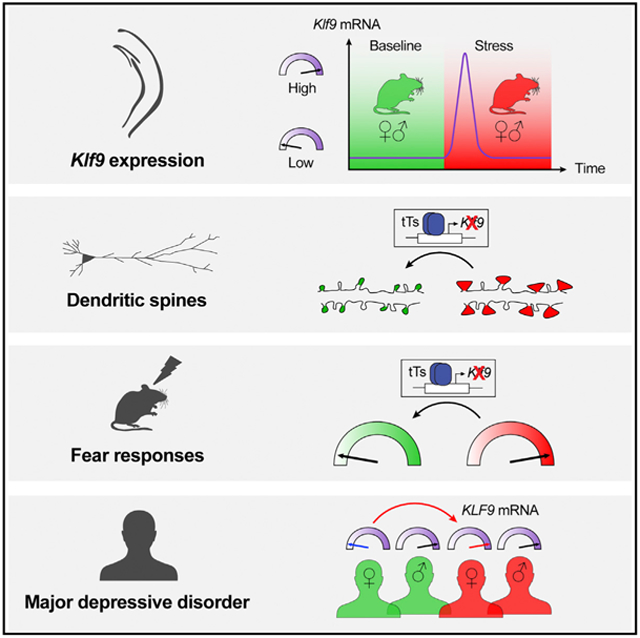

## INTRODUCTION

It is widely recognized that post-traumatic stress disorder (PTSD) and major depressive disorder (MDD) arise from interactions between environmental factors such as stressful, traumatic life events and genetic risk factors (Besnard and Sahay, 2016; Caspi et al., 2010; Caspi and Moffitt, 2006; Liberzon and Abelson, 2016; Shin and Liberzon, 2010; Smoller, 2016). Furthermore, the incidence of stress-related psychopathologies including MDD and PTSD is higher in women than in men (Haskell et al., 2010; Kessler et al., 1993,^2005^; Kornstein et al., 2000). Traumatic and stressful life events activate the hypothalamic-pituitary axis and trigger release of nuclear stress hormones and glucocorticoids or recruit other physiological neuropeptide effectors (de Kloet et al., 2005) that modify neural circuits (McEwen et al., 2016). Sustained elevation of glucocorticoids or prolonged exposure to restraint stress is thought to induce maladaptive remodeling in the hippocampus (and other brain regions) and, consequently, to impair contextual fear memory processing and regulation of the hypothalamic-pituitary axis (HPA). At a neuronal level, there is growing appreciation that dendritic spines in different cell types respond differentially to chronic stressors (Gourley et al., 2013; Liston and Gan, 2011; Sousa et al., 2000; Yau et al., 2016). Glucocorticoids increase dendritic spine size on shorter timescales and may increase or decrease dendritic spine density depending on duration of exposure and cell type (Liston et al., 2013; Liston and Gan, 2011; Russo et al., 2016). Social defeat stress, on the other hand, increases the number of stubby excitatory spines (Christoffel et al., 2011). Together, these observations suggest that chronic stressors signal to the cytoskeleton, via the nucleus or non-genomically, to modify dendritic spines. However, despite advances in identifying transcription factors that sense chronic stress signals to orchestrate changes in excitability (Gray et al., 2017; Han and Nestler, 2017; Harris et al., 2015), we know remarkably less about stress-responsive transcription factors that regulate dendritic spine remodeling in the hippocampus (Mucha et al., 2011; Son et al., 2012).

Previous work has suggested that Kruppel-like factor 9 (*Klf9*) is a negative transcriptional regulator of dendritic spines (McAvoy et al., 2016) in stress-naive mice and is a primary transcriptional target of glucocorticoids (Bagamasbad et al., 2012; Bonett et al., 2009; Datson et al., 2011). Acute, but not chronic, corticosterone administration elevates *Klf9* expression in the hippocampus of male mice (Carter et al., 2013) and drives glucocorticoid receptor occupancy at glucocorticoid response elements within the *Klf9* promoter in dentate gyrus (DG) of male rats (Bagamasbad et al., 2012). Consistent with these findings, *KLF9* expression is upregulated in the DG of individuals with MDD (Duric et al., 2010). These observations suggest that *Klf9* may be a stress-sensitive factor that constrains maladaptive dendritic spine remodeling in the hippocampus and fear responses.

Here we show in rodents that acute restraint stress, but not chronic restraint stress (CRS), increased expression of *Klf9* in hippocampus. To determine whether CRS- and corticosterone (CORT)-induced transient *Klf9* upregulation in the hippocampus is adaptive or maladaptive, we genetically engineered mice in which we could inducibly silence *Klf9* expression in excitatory forebrain neurons. We found that inducible genetic silencing of *Klf9* in excitatory forebrain neurons of stress-naive adult male or female mice did not affect contextual fear processing at baseline. However, *Klf9* silencing prevented CRS-induced potentiation of contextual fear acquisition in female mice and chronic CORT exposure-induced generalization of fear in male mice. Interestingly, these protective effects were seen only when *Klf9* was silenced prior to, but not after, the onset of the stressor. At a circuit level, we found that both chronic CORT and CRS induced enlargement of dendritic spines in the ventral hippocampus in males and females, respectively, and that silencing of *Klf9* expression prevented these alterations. Analysis of human postmortem hippocampal tissue revealed increased *KLF9* mRNA density in the anterior DG of women, but not men, with more severe recent stressful life events and increased mortality (suicide). Together, these observations implicate *Klf9* as a stress-responsive transcription factor and demonstrate that targeting *Klf9* is sufficient to confer resilience to chronic stress-induced enlargement of dendritic spines in ventral hippocampus and maladaptive fear responses in a sex-specific manner.

## RESULTS

### CRS Decreases *Klf9* Expression in Forebrain Circuits

**Figure 1.**
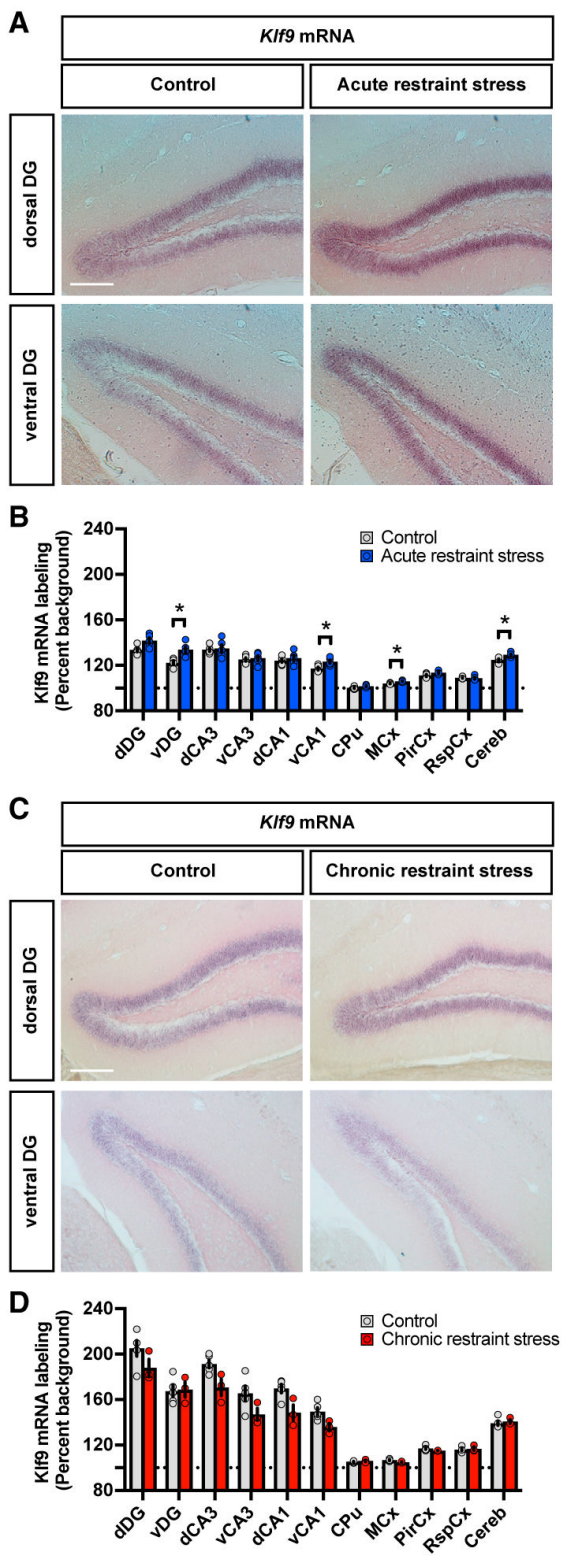
Chronic Restraint Stress Decreases *Klf9* Expression in Forebrain Circuits (A) *In situ* hybridization using specific riboprobes to assess Kruppel-like factor 9 (*Klf9*) mRNA expression in the hippocampus of mice acutely restrained for 6 hr (right) and home cage controls (left). Representative images for five independent animals per group. Scale bar: 200 μm. (B) Quantifications of *Klf9* mRNA expression (percentage background) across the hippocampus, cortical areas, and cerebellum. Data (mean ± SEM; n = 5 and 5 mice per group) were analyzed using unpaired two-tailed Student’s t test; *p < 0.05, acute stress versus controls. (C) *In situ* hybridization of *Klf9* mRNA expression in the hippocampus of mice chronically (10 days) restrained for 6 hr (right) and home cage controls (left). Representative images for five and three independent animals per group. Scale bar: 200 μm. (D) Quantifications of *Klf9* mRNA expression (percentage background) across the hippocampus, cortical areas, and cerebellum. Data (mean ± SEM; n = 5 and 3 mice per group) were analyzed using unpaired two-tailed Student’s t test.

Studies in rodents and *Xenopus* suggest a role for acute stress (glucocorticoid exposure or handling stress) in elevating *Klf9* expression levels (Bagamasbad et al., 2012; Bonett et al., 2009; Datson et al., 2011). Furthermore, preliminary microarray analysis of postmortem hippocampal tissue of MDD patients reported elevated *KLF9* expression in the DG, although characterization of *KLF9* expression as a function of gender, stress history of subjects, and anterior-posterior axis of DG was not assessed (Duric et al., 2010). In order to gain further insights into the relationship between stress (acute versus chronic) and *Klf9* expression, we analyzed the pattern of *Klf9* expression in mice subjected to acute restraint stress (6 hr, 1 day) and CRS (6 hr, 10 days). Upon completion of the restraint stress procedure, mice were sacrificed and the brains were processed for *in situ* hybridization studies using a riboprobe specific for *Klf9* (Scobie et al., 2009). We found that acute restraint stress, like acute glucocorticoid exposure, increased *Klf9* expression levels in ventral DG and ventral CA1, motor cortex and cerebellum (Figures 1A and 1B). In contrast, *Klf9* expression was not elevated in these areas following CRS (Figures 1C and 1D). Interestingly, chronic CORT treatment, unlike acute CORT treatment, does not elevate *Klf9* expression in the hippocampus (Carter et al., 2013).

### A Genetic System to Inducibly and Reversibly Silence *Klf9* Expression in Excitatory Forebrain Neurons

**Figure 2.**
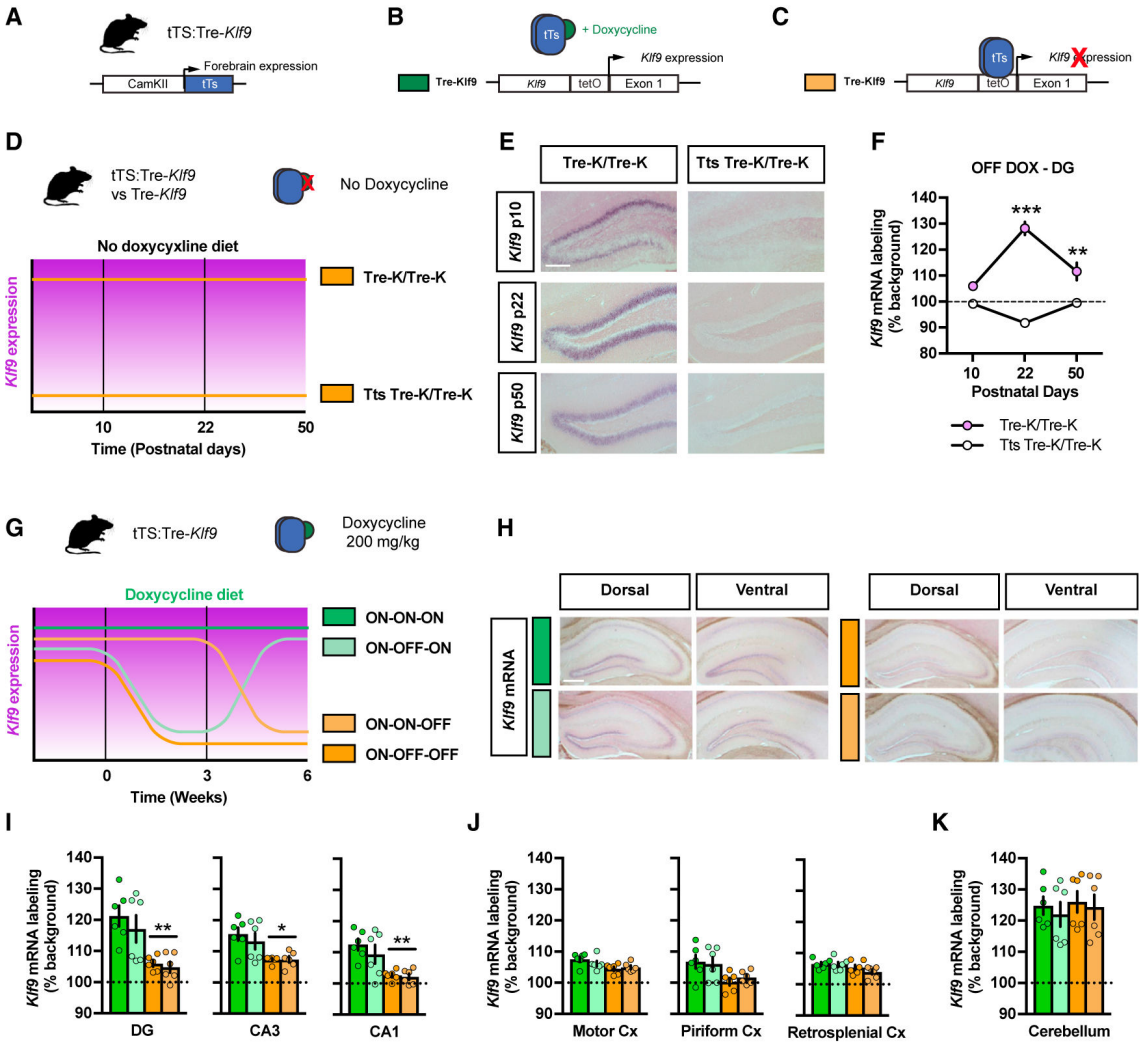
A Genetic System to Inducibly and Reversibly Silence *Klf9* Expression in Excitatory Forebrain Neurons (A–C) Schematic representation of mice homozygous for Tre-*Klf9* and expressing tTS in the forebrain under the control of a CamKIIα promoter (A). This genetic system is responsive to doxycycline, which is provided in the animal’s diet. Doxycycline diet (B) or withdrawal (C) prevents or triggers the silencing of *Klf9*. (D) Schematic representation for Tre-*Klf9*/CamKIIα-tTS mice (referred to as tTS:Tre-*Klf9*) and Tre-*Klf9* (no CamKIIα-tTS) mice raised without doxycycline (OFF) for 10, 22, and 50 postnatal days. (E) *In situ* hybridization of *Klf9* mRNA expression in the dentate gyrus (DG) of Tre-*Klf9* (left) and tTS:Tre-*Klf9* (right). Representative images for three independent animals per group. Scale bar: 200 μm. (F) Quantifications of *Klf9* mRNA expression (percentage background) in the DG. Data (mean ± SEM; n = 3, 3, 3, 3, 3, and 3 mice per group) were analyzed using mixed-factor two-way ANOVA: time F_(2,12)_ = 8.33, p < 0.01; doxycycline F_(1,12)_ = 152.12, p < 0.001; interaction F_(2,12)_ = 37.18, p < 0.001. **p < 0.01 and ***p < 0.001, tTS:Tre-*Klf9* versus Tre-*Klf9*. (G) Schematic representation of tTS:Tre-*Klf9* mice with different doxycycline diet schedules. (H) *In situ* hybridization of *Klf9* mRNA expression in the DG of tTS:Tre-*Klf9* with different doxycycline diet schedules. Representative images for six independent animals per group. Scale bar: 200 μm. (I–K) Quantifications of *Klf9* mRNA expression (percentage background) in the hippocampus (I), cortices (J), and cerebellum (K). Data (mean ± SEM; n = 6, 6, 6, and 6 mice per group) were analyzed using one-way ANOVA followed by Tukey’s multiple-comparisons post hoc test. *p < 0.05 and **p < 0.01, OFF DOX versus ON DOX.

To determine whether the transient *Klf9* upregulation seen following onset of stressor is adaptive or maladaptive, we engineered mice in which we can inducibly silence *Klf9* expression in distinct cell types. *Klf9* is expressed in neuronal and nonneuronal cell types (Carter et al., 2013; Dugas et al., 2012; McAvoy et al., 2016; Scobie et al., 2009), and we chose to target *Klf9* expression in excitatory neurons, as the glucocorticoid receptor is required in excitatory forebrain neurons for modulating fear responses (Hartmann et al., 2017). Toward this goal, we bred mice that we engineered to carry a tetracycline response element (Tre) knocked in upstream of the *Klf9* gene (*Tre-Klf9*) (McAvoy et al., 2016) with mice carrying a tetracycline-controlled transcriptional silencer (tTS) transgene under the control of a CamKIIα promoter fragment (Richardson-Jones et al., 2011) (Figure 2A). Doxycycline (DOX) in the diet (Figure 2B) or withdrawal (Figure 2C) prevents or triggers the silencing of the gene of interest (*Klf9*), respectively, in bigenic mice carrying two copies of the *Tre-Klf9* allele and one copy of the tTS transgene (referred to as tTS:Tre-*Klf9*). We first analyzed *Klf9* expression in tTS:Tre-*Klf9* mice and control littermates lacking the *tTS* transgene (Tre-*Klf9*) that were raised without DOX (OFF) and perfused at 10, 22, and 50 days following birth (Figure 2D). *In situ* hybridization analysis revealed that CamKIIα-tTS potently suppresses *Klf9* expression in DG and other hippocampal subregions (Figures 2E and 2F; data not shown). We then asked whether we could use our bigenic system to inducibly and reversibly silence *Klf9* expression in the forebrain. We generated four groups of adult tTS:Tre-*Klf9* mice raised on DOX in diet until 2 months of age, following which two groups were maintained on DOX (ON) and two groups were taken off DOX (OFF) for 3 weeks (0 week time point) (Figure 2G). One of the ON groups was then taken off DOX to assess inducible silencing (ON-ON-OFF) and the other one maintained on DOX to permit assessment of leakiness (ON-ON-ON). One of the OFF groups was brought back on DOX to determine if silencing was reversible (ON-OFF-ON), while the other group remained off DOX to permit examination of sustained inducible silencing (ON-OFF-OFF). We then examined *Klf9* expression levels in the hippocampus, cortex (motor, piriform, and retrosplenial), and cerebellum (Figure 2G). Our results suggest that DOX administration conferred inducible silencing of *Klf9* expression in tTS:Tre-*Klf9* mice. Specifically, ON-ON-OFF and ON-OFF-OFF mice showed significantly lower *Klf9* expression in DG, CA3 and CA1 compared with ON-ON-ON and ON-OFF-ON mice (Figures 2H and 2I). Furthermore, silencing of *Klf9* expression was reversible as evidenced in the ON-OFF-ON and ON-ON-ON groups that exhibited similar levels of *Klf9* expression (Figures 2H and 2I). These effects were detected in the hippocampus and not observed in the cortices examined and cerebellum (Figures 2J and 2K). Together, these findings demonstrate the utility of tTS:Tre-*Klf9* mice in inducible and reversible silencing of *Klf9* expression in the hippocampus.

### CRS and Chronic CORT Treatment Differentially Affect Fear Responses in Male and Female Mice

Growing evidence suggests that male and female mice respond differentially to stressors (Gray et al., 2017). Therefore, we asked whether chronic CORT and CRS differentially affect contextual fear memory in male and female tTS:Tre-*Klf9* mice with intact levels of *Klf9* expression (maintained on DOX) (Figure S1). Upon exposure to 5 weeks of 35 μg/mL CORT, male tTS:Tre-*Klf9* mice (Figure S1A) showed normal contextual fear conditioning (CFC) in context A (Figures S1B and S1D) but increased fear generalization in a distinct context C (Figure S1E). Conversely, female tTS:Tre-*Klf9* mice (Figure S1A) showed no alteration in contextual fear learning and generalization in response to 35 μg/mL CORT treatment (Figures S1F and S1G). These results are in line with recent findings suggesting that chronic CORT exposure has limited effects on behavioral emotionality in female mice (Mekiri et al., 2017). Furthermore, male but not female mice exhibited CORT-dependent weight gain (data not shown). In contrast to CORT, 2 weeks of CRS potentiated contextual fear acquisition in female but not male tTS:Tre-*Klf9* mice (Figures S1H-S1N).

### Inducible Silencing of *Klf9* Expression in Forebrain Excitatory Neurons Prevents Chronic CORT-Induced Overgeneralization of Contextual Fear in Male Mice

**Figure 3.**
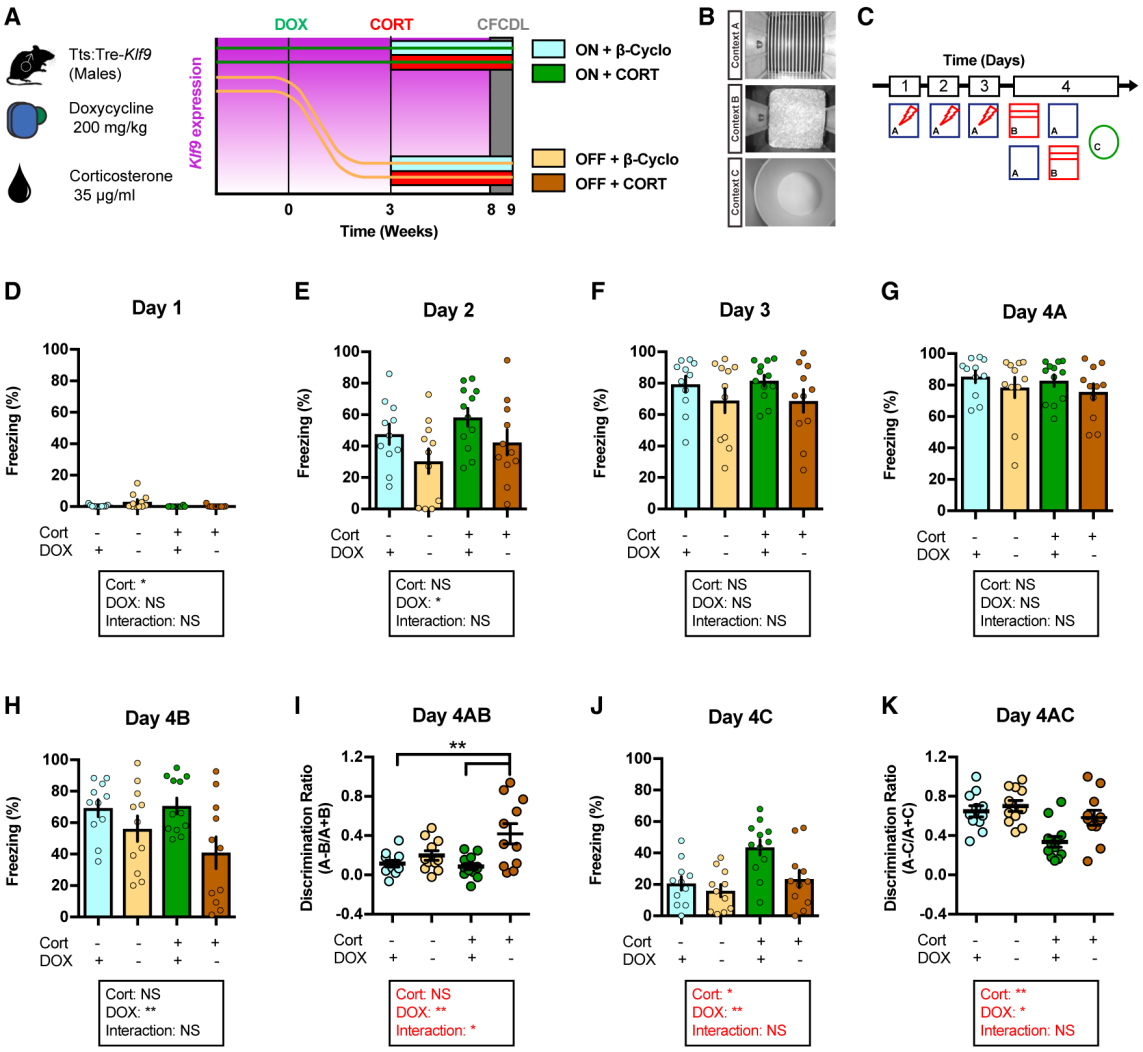
Inducible Silencing of *Klf9* Expression in Forebrain Excitatory Neurons of Male Mice Prevents Chronic CORT-Induced Overgeneralization of Contextual Fear (A) Schematic representation of tTS:Tre-*Klf9* male mice with different doxycycline and corticosterone diet schedules. (B) CFCDL procedure in which mice were trained to discriminate between a footshock delivered in context A and safe contexts B and C. (C) Schematic representation of CFCDL timeline consisting of 3 days of training in context A and discrimination tests on day 4. (D) Freezing behavior on day 1. Data (mean ± SEM; n = 11, 11, 12, and 11 mice per group) were analyzed using mixed-factor two-way ANOVA: corticosterone F_(1,41)_ = 5.32, p < 0.05; doxycycline F_(1,41)_ = 3.71, not significant (NS); interaction F_(1,41)_ = 2.96, NS. (E) Freezing behavior on day 2. Data (mean ± SEM; n = 11, 11, 12, and 11 mice per group) were analyzed using mixed-factor two-way ANOVA: corticosterone F_(1,41)_ = 2.65, NS; doxycycline F_(1,41)_ = 5.63, p < 0.05; interaction F_(1,41)_ = 0.00, NS. (F) Freezing behavior on day 3. Data (mean ± SEM; n = 11, 11, 12, and 11 mice per group) were analyzed using mixed-factor two-way ANOVA: corticosterone F_(1,41)_ = 0.02, NS; doxycycline F_(1,41)_ = 3.69, NS; interaction F_(1,41)_ = 0.03, NS. (G) Freezing behavior on day 4A. Data (mean ± SEM; n = 11, 11, 12, and 11 mice per group) were analyzed using mixed-factor two-way ANOVA: corticosterone F_(1,41)_ = 0.29, NS; doxycycline F_(1,41)_ = 2.18, NS; interaction F_(1,41)_ = 0.00, NS. (H) Freezing behavior on day 4B. Data (mean ± SEM; n = 11, 11, 12, and 11 mice per group) were analyzed using mixed-factor two-way ANOVA: corticosterone F_(1,41)_ = 0.88, NS; doxycycline F_(1,41)_ = 8.53, p < 0.01; interaction F_(1,41)_ = 1.29, NS. (I) Discrimination ratio calculated for contexts A and B. Data (mean ± SEM; n = 11, 11, 12, and 11 mice per group) were analyzed using mixed-factor two-way ANOVA: corticosterone F_(1,41)_ = 2.49, NS; doxycycline F_(1,41)_ = 11.67, p < 0.01; interaction F_(1,41)_ = p < 0.05; **p < 0.01, ON CORT OFF DOX versus ON DOX. (J) Freezing behavior on day 4C. Data (mean ± SEM; n = 11, 11, 12, and 11 mice per group) were analyzed using mixed-factor two-way ANOVA: corticosterone F_(1,41)_ = 10.34, p < 0.01; doxycycline F_(1,41)_ = 6.88, p < 0.05; interaction F_(1,41)_ = 2.67, NS. (K) Discrimination ratio calculated for contexts A and C. Data (mean ± SEM; n = 11, 11, 12, and 11 mice per group) were analyzed using mixed-factor two-way ANOVA: corticosterone F_(1,41)_ = 12.52, p < 0.01; doxycycline F_(1,41)_ = 6.01, p < 0.05; interaction F_(1,41)_ = 2.44, NS. See also Figures S1 and S2.

We next sought to leverage the tTS:Tre-*Klf9* mice to causally ascertain whether silencing *Klf9* expression protects against chronic CORT-induced fear responses in male mice (Figure 3A). Four groups of male tTS:Tre-*Klf9* mice were maintained on DOX until 2 months of age. Two groups were then kept on DOX and two groups were switched off DOX for 3 weeks, following which each pair of ON and OFF DOX groups was exposed to either chronic CORT (stress condition) or β-cyclodextrin vehicle in drinking water while remaining on or off DOX, respectively (non-stress condition) (Figure 3A). We examined CFC in context A, discrimination between the training context A and a safe, similar context B, and generalization in a distinct context C 5 weeks after the initiation of CORT or β-cyclodextrin treatment (Figure 3B). Unlike *Klf9*-null mice that lack *Klf9* in all cell types and throughout life (Scobie et al., 2009), stress-naive mice in which *Klf9* expression was acutely silenced in excitatory forebrain neurons did not exhibit impairments in CFC, discrimination, or generalization in the absence of stress (Figures 3C-3K). In contrast, inducible *Klf9* silencing in excitatory forebrain neurons prevented CORT-induced generalization of fear in context C (Figures 3C-3K). Specifically, CORT-treated mice showed enhanced levels of freezing compared with vehicle-treated mice in the safe, distinct context C (Figure 3J). This effect was completely reversed in CORT-treated mice, in which *Klf9* expression was silenced in excitatory hippocampal neurons (OFF DOX) (Figure 3J).

Next, we asked whether silencing *Klf9* expression following acute CORT-dependent elevation in *Klf9* expression prevents CORT-induced fear overgeneralization in male mice (Figure S2A). Tre-*Klf9* and tTS:Tre-*Klf9* male mice were maintained on DOX until 2 months of age, and then both groups were exposed to CORT in drinking water for 1 week to induce elevation in *Klf9* expression. After 1 week of CORT treatment and with CORT on board, we silenced *Klf9* expression by removal of DOX for 4.5 weeks and tested the mice in CFC, context discrimination and generalization assays (Figures S2B and S2C). Interestingly, unlike mice in which *Klf9* expression was silenced prior to chronic CORT treatment (Figure 3), silencing *Klf9* expression following 1 week of CORT treatment did not prevent CORT-induced fear overgeneralization (Figures S2D-S2F).

Collectively, these results indicate that decreasing *Klf9* expression in excitatory forebrain neurons prevents CORT-induced fear overgeneralization in male mice. Furthermore, *Klf9* silencing in excitatory forebrain neurons after the acute phase of CORT exposure is not sufficient to prevent CORT-induced fear overgeneralization in male mice.

### Inducible Silencing of *Klf9* Expression in Forebrain Excitatory Neurons Prevents Chronic CORT-Induced Enlargement in Dendritic Spines in Ventral DG in Male Mice

**Figure 4.**
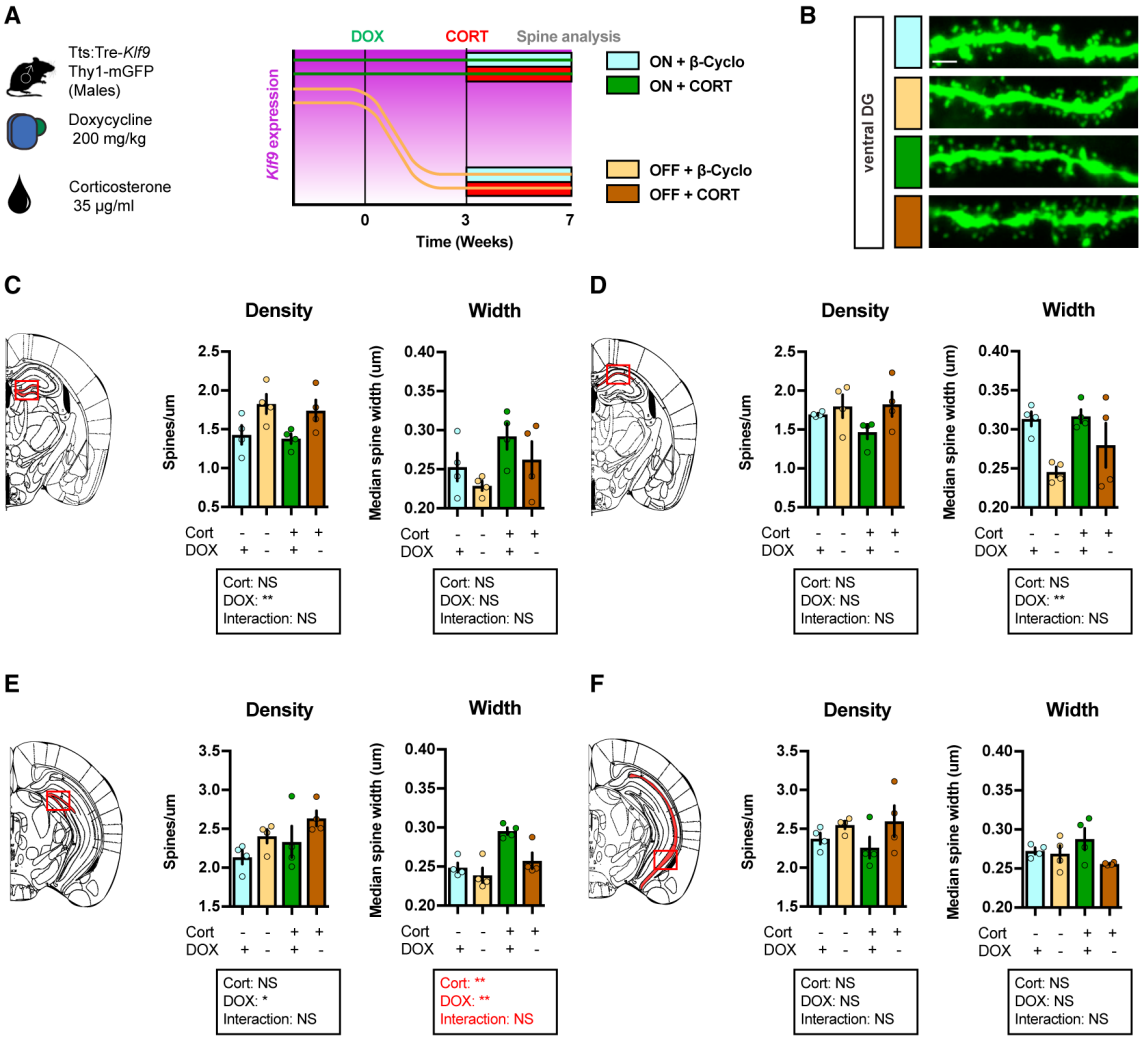
Inducible Silencing of *Klf9* Expression in Forebrain Excitatory Neurons of Male Mice Prevents Chronic CORT-Induced Enlargement in Dendritic Spines in Ventral DG (A) Schematic representation of tTS:Tre-*Klf9*:Thy1-GFP/+ male mice with different doxycycline and corticosterone diet schedules. (B) Maximum intensity projection confocal images of individual dendritic segments from the outer molecular layer in ventral DG. Representative images for four independent animals per group. Scale bar: 2 μm. (C) Spine density and width in dorsal DG (red box). Data (mean ± SEM; n = 4, 4, 4, and 4 mice per group) were analyzed using mixed-factor two-way ANOVA: density: corticosterone F_(1,12)_ = 0.34, NS; doxycycline F_(1,12)_ = 11.0, p < 0.01; interaction F_(1,12)_ = 0.02, NS; width: corticosterone F_(1,12)_ = 4.56, NS; doxycycline F_(1,12)_ = 2.45, NS; interaction F_(1,12)_ = 0.02, NS. (D) Spine density and width in dorsal CA1 (red box). Data (mean ± SEM; n = 4, 4, 4, and 4 mice per group) were analyzed using mixed-factor two-way ANOVA: density: corticosterone F_(1,12)_ = 0.75, NS; doxycycline F_(1,12)_ = 3.91, NS; interaction F_(1,12)_ = 1.18, NS; width: corticosterone F_(1,12)_ = 1.42, NS; doxycycline F_(1,12)_ = 10.99, p < 0.01; interaction F_(1,12)_ = 0.94, NS. (E) Spine density and width in ventral DG (red box). Data (mean ± SEM; n = 4, 4, 4, and 4 mice per group) were analyzed using mixed-factor two-way ANOVA: density: corticosterone F_(1,12)_ = 2.78, NS; doxycycline F_(1,12)_ = 4.97, p < 0.05; interaction F_(1,12)_ = 0.01, NS; width: corticosterone F_(1,12)_ = 17.59, p < 0.01; doxycycline F_(1,12)_ = 9.34, p < 0.01; interaction F_(1,12)_ = 3.29, NS. (F) Spine density and width in ventral CA1 (red box). Data (mean ± SEM; n = 4, 4, 4, and 4 mice per group) were analyzed using mixed-factor two-way ANOVA: density: corticosterone F_(1,12)_ = 0.07, NS; doxycycline F_(1,12)_ = 3.99, NS; interaction F_(1,12)_ = 0.41, NS; width: corticosterone F_(1,12)_ = 0.01, NS; doxycycline F_(1,12)_ = 3.97, NS; interaction F_(1,12)_ = 2.55, NS.

Because both stress and Klf9 target dendritic spines, we next examined whether *Klf9* silencing could reverse CORT-induced changes to hippocampal spine morphology. We thus examined dendritic spines in DG and CA1 of dorsal and ventral hippocampus in non-stressed and chronic CORT-treated triple transgenic male mice tTS:Tre-*Klf9*:Thy1-GFP/+ in which the Thy-1 GFP allele permits evaluation of dendritic spine structure and density (Figures 4A and 4B). Silencing *Klf9* expression increased dendritic spine density in dorsal and ventral DG, but not CA1, of non-stressed mice (Figures 4C and 4E). Interestingly, and consistent with previous reports (Sousa et al., 2000; Yau et al., 2016), chronic CORT did not affect dendritic spine density in DG and CA1 (Figures 4C-4F). However, analysis of dendritic spine width revealed that CORT induced an enlargement of spines in ventral DG and that *Klf9* silencing reversed this spine enlargement phenotype (Figure 4E). Together, these observations suggest that targeting CORT-induced increase in dendritic spine width in ventral DG may represent a potential substrate for preventing CORT-induced fear overgeneralization.

### Inducible Silencing of *Klf9* Expression in Forebrain Excitatory Neurons Prevents CRS-Induced Potentiation of Contextual Fear Acquisition in Female Mice

**Figure 5.**
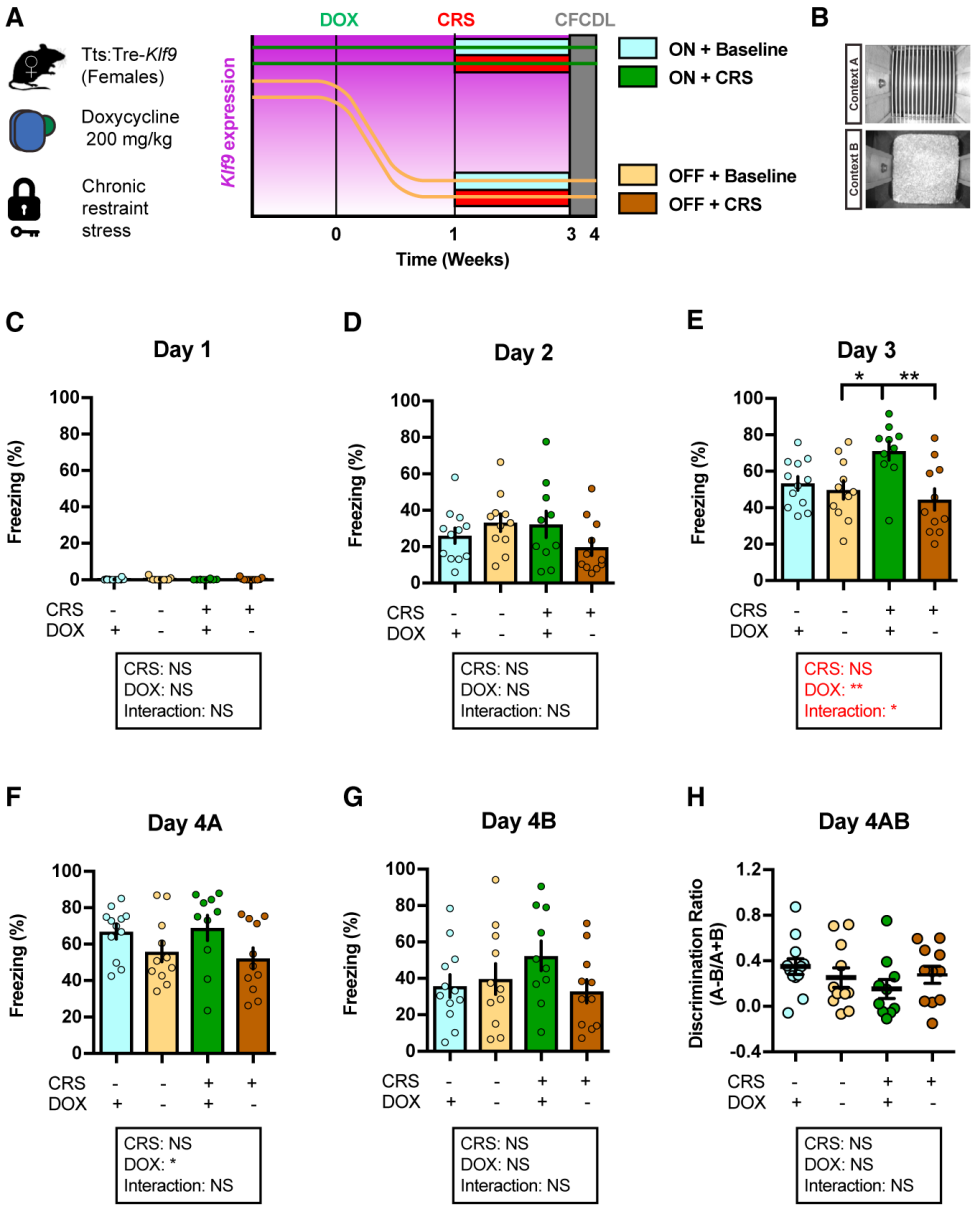
Inducible Silencing of *Klf9* Expression in Forebrain Excitatory Neurons of Female Mice Prevents CRS-Induced Potentiation of Contextual Fear Acquisition (A) Schematic representation of tTS:Tre-*Klf9* female mice with different doxycycline diet schedules and chronic restraint stress timeline. (B) CFCDL procedure in which mice were trained to discriminate between a footshock delivered in context A and safe context B. (C) Freezing behavior on day 1. Data (mean ± SEM; n = 12, 11, 10, and 11 mice per group) were analyzed using mixed-factor two-way ANOVA: CRS F_(1,40)_ = 0.13, NS; doxycycline F_(1,40)_ = 0.97, NS; interaction F_(1,40)_ = 0.04, NS. (D) Freezing behavior on day 2. Data (mean ± SEM; n = 12, 11, 10, and 11 mice per group) were analyzed using mixed-factor two-way ANOVA: CRS F_(1,40)_ = 0.49, NS; doxycycline F_(1,40)_ = 0.28, NS; interaction F_(1,40)_ = 3.64, NS. (E) Freezing behavior on day 3. Data (mean ± SEM; n = 12, 11, 10, and 11 mice per group) were analyzed using mixed-factor two-way ANOVA: CRS F_(1,40)_ = 1.62, NS; doxycycline F_(1,40)_ = 9.35, p < 0.01; interaction F_(1,40)_ = 5.4, p < 0.05; *p < 0.05 and **p < 0.01, ON CORT ON DOX versus OFF DOX. (F) Freezing behavior on day 4A. Data (mean ± SEM; n = 12, 11, 10, and 11 mice per group) were analyzed using mixed-factor two-way ANOVA: CRS F_(1,40)_ = 0.01, NS; doxycycline F_(1,40)_ = 6.29, p < 0.05; interaction F_(1,40)_ = 0.27, NS. (G) Freezing behavior on day 4B. Data (mean ± SEM; n = 12, 11, 10, and 11 mice per group) were analyzed using mixed-factor two-way ANOVA: CRS F_(1,40)_ = 0.46, NS; doxycycline F_(1,40)_ = 1.14, NS; interaction F_(1,40)_ = 2.56, NS. (H) Discrimination ratio calculated for contexts A and B. Data (mean ± SEM; n = 12, 11, 10, and 11 mice per group) were analyzed using mixed-factor two-way ANOVA: CRS F_(1,40)_ = 1.16, NS; doxycycline F_(1,40)_ = 0.03, NS; interaction F_(1,40)_ = 1.99, NS. See also Figures S1 and S2.

Our analysis of chronic stressors in male and female mice revealed that CRS potentiates contextual fear acquisition in female, but not male, mice (Figures S1K-S1N). Therefore, we next asked whether inducible *Klf9* silencing is protective against the effects of another chronic stressor, CRS, on fear responses in female tTS:Tre-*Klf9* mice (Figure 5A). To this end, we used four groups of female tTS:Tre-*Klf9* that were maintained on DOX until 2 months of age. We then took two groups off DOX for 1 week prior to initiating CRS for one pair of ON and OFF DOX groups for 14 days (stress condition) while the other pair of ON and OFF DOX groups remained in the home cage (non-stress condition) (Figure 5A). Ten days following cessation of CRS, we examined CFC in context A and discrimination between the training context A and a safe, similar context B (Figure 5B). *Klf9* silencing in forebrain excitatory neurons prevented CRS-induced potentiation of contextual fear acquisition without affecting fear responses in non-stressed mice (Figures 5C-5E). Additionally, inducible *Klf9* silencing in stressed and non-stressed groups did not affect contextual fear discrimination as assessed by measuring freezing levels in contexts A and B (Figures 5F-5H).

Next, we asked whether silencing *Klf9* expression following acute restraint stress (rather than prior) protects against CRS-induced potentiation of fear responses in female mice (Figure S2G). Two groups of female Tre-*Klf9* and tTS:Tre-*Klf9* mice were maintained on DOX until 2 months of age, and then both groups were subjected to CRS for 10 days. Following cessation of CRS, both groups were taken off DOX for 2 weeks and tested in CFC, context discrimination, and generalization assays (Figures S2H and S2I). Unlike the mice in which *Klf9* expression was decreased prior to and during CRS (Figure 5), silencing *Klf9* expression following CRS did not reverse CRS-induced potentiation of fear responses (Figures S2J-S2L).

Taken together with our studies using CORT, these results suggest that decreasing *Klf9* expression prior to (and during) the onset of stressor protects against stress-induced maladaptive fear responses.

### Inducible Silencing of *Klf9* Expression in Forebrain Excitatory Neurons Prevents CRS Enlargement in Dendritic Spines in Ventral CA1 in Female Mice

**Figure 6.**
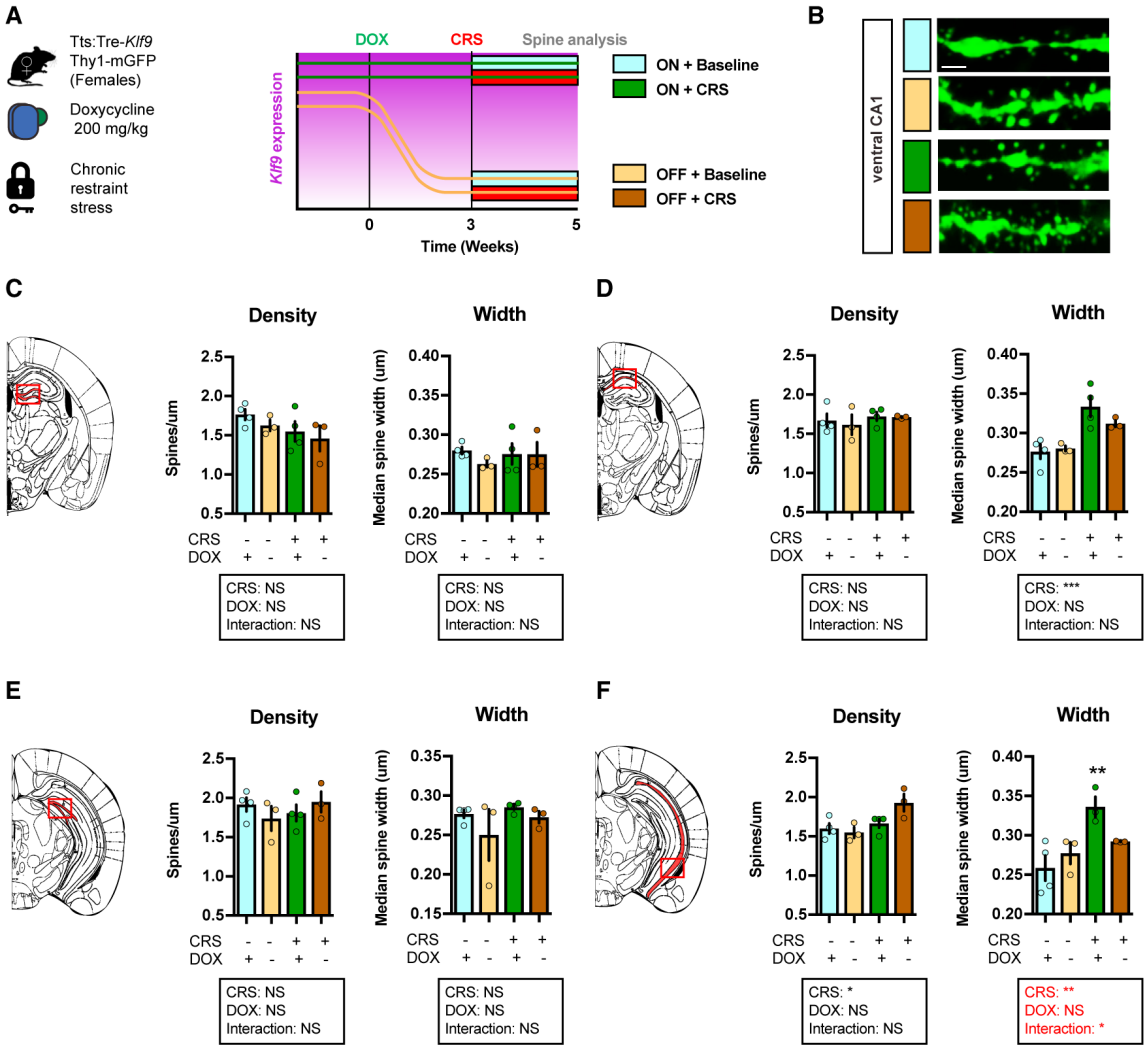
Inducible Silencing of *Klf9* Expression in Forebrain Excitatory Neurons of Female Mice Prevents CRS Enlargement in Dendritic Spines in Ventral CA1 (A) Schematic representation of tTS:Tre-*Klf9*:Thy1-GFP/+ female mice with different doxycycline diet schedules and chronic restraint stress timeline. (B) Maximum intensity projection confocal images of individual dendritic segments from the stratum radiatum in ventral CA1. Representative images for four independent animals per group. Scale bar: 2 μm. (C) Spine density and width in dorsal DG (red box). Data (mean ± SEM; n = 4, 3, 4, and 3 mice per group) were analyzed using mixed-factor two-way ANOVA: density: CRS F_(1,10)_ = 2.86, NS; doxycycline F_(1,10)_ = 1.03, NS; interaction F_(1,10)_ = 0.04, NS; width: CRS F_(1,10)_ = 0.13, NS; doxycycline F_(1,10)_ = 0.68, NS; interaction F_(1,10)_ = 0.6, NS. (D) Spine density and width in dorsal CA1 (red box). Data (mean ± SEM; n = 4, 3, 4, and 3 mice per group) were analyzed using mixed-factor two-way ANOVA: density: CRS F_(1,10)_ = 0.87, NS; doxycycline F_(1,10)_ = 0.15, NS; interaction F_(1,10)_ = 0.08, NS; width: CRS F_(1,10)_ = 21.42, p < 0.001; doxycycline F_(1,10)_ = 0.82, NS; interaction F_(1,10)_ = 1.76, NS. (E) Spine density and width in ventral DG (red box). Data (mean ± SEM; n = 4, 3, 4, and 3 mice per group) were analyzed using mixed-factor two-way ANOVA: density: CRS F_(1,10)_ = 0.19, NS; doxycycline F_(1,10)_ = 0.02, NS; interaction F_(1,10)_ = 1.85, NS; width: CRS F_(1,10)_ = 1.16, NS; doxycycline F_(1,10)_ = 1.88, NS; interaction F_(1,10)_ = 0.23, NS. (F) Spine density and width in ventral CA1 (red box). Data (mean ± SEM; n = 4, 3, 4, and 3 mice per group) were analyzed using mixed-factor two-way ANOVA: density: CRS F_(1,10)_ = 9.19, p < 0.05; doxycycline F_(1,10)_ = 2.08, NS; interaction F_(1,10)_ = 4.6, NS; width: CRS F_(1,9)_ = 11.88, p < 0.01; doxycycline F_(1,9)_ = 0.87, NS; interaction F_(1,9)_ = 5.48, p < 0.05; *p < 0.05 and **p < 0.01, ON CORT ON DOX versus OFF CORT ON DOX. See also Figure S3.

We next sought to determine the effects of inducible *Klf9* silencing on CRS-induced alterations in dendritic spines in DG and CA1 using female triple transgenic tTS:Tre-*Klf9*:Thy1-GFP/ + mice (Figure 6A). CRS and inducible *Klf9* silencing did not affect dendritic spine density in DG and CA1 (Figures 6B-6F). Remarkably, CRS increased dendritic spine width in CA1, but not DG, and this effect was completely reversed by *Klf9* silencing in ventral CA1 (Figure 6F). Furthermore, CRS failed to enlarge dendritic spines in male triple transgenic tTS:Tre-*Klf9*:Thy1-GFP/+ mice (Figure S3). These results suggest ventral CA1 as a potential substrate for the protective effects of *Klf9* silencing on CRS-induced potentiation of contextual fear acquisition in female mice.

### Increased *KLF9* Expression in the DG of Women with MDD-Suicide and Recent History of Stressful Life Events

**Figure 7.**
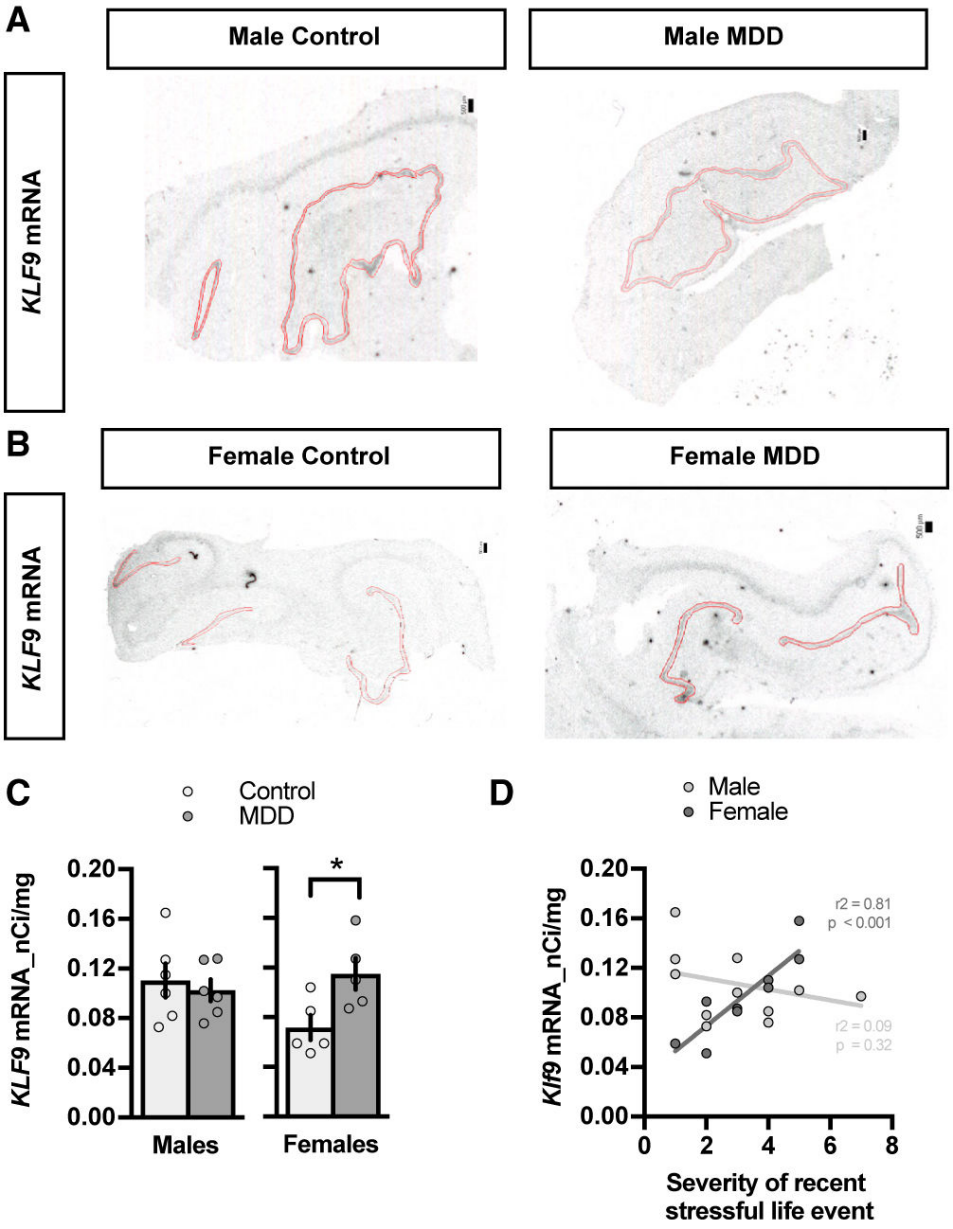
Increased *KLF9* Expression in the DG of Women with Recent History of Stressful Life Events (A and B) Post mortem *in situ* hybridization using specific riboprobes to assess *KLF9* mRNA expression in the anterior DG (red outline) of male (A) and female (B) human subjects with recent history of stressful life events and major depressive disorder (MDD). Representative images for six, six, five, and five independent subjects per group. Scale bar: 50 μm. (C) Quantifications of *KLF9* mRNA expression (percentage background) in the anterior DG of male and female subjects. Data (mean ± SEM; n = 6 and 6 subjects per group) were analyzed using unpaired two-tailed Student’s t test. Data (mean ± SEM; n = 5 and 5 subjects per group) were analyzed using unpaired two-tailed Student’s t test; *p < 0.05, MDD versus controls. (D) Quantifications of *KLF9* mRNA expression (percentage background) in the anterior DG correlated with the severity of recent stressful life events. Data (n = 10 and 12 subjects per group). Note the significant positive correlation between the *KLF9* mRNA levels and the severity of recent stressful life events in female (r^2^ = 0.818, p = 0.0003) but not male (r^2^ = 0.095, p = 0.328) subjects. See also Table S1.

A microarray analysis performed on postmortem hippocampal tissue of individuals with MDD found *KLF9* expression to be elevated in the DG (Duric et al., 2010). However, the study did not assess *KLF9* expression in relation to recent or chronic stress, sex, and subject treatment status. Moreover, *KLF9* expression was not examined along the anterior-posterior axis of DG. Approximately 40% of subjects with MDD had prescriptions for antidepressants filled in the last month of life, and compliance was unknown, but ongoing antidepressant treatment could potentially affect *KLF9* mRNA expression. To begin to better understand how *KLF9* expression is affected by recent life event-related stress exposure and gender, we performed *in situ* hybridization on postmortem hippocampus from 12 subjects with untreated MDD, 10 of whom died by suicide, and 12 controls with no history of neuropsychiatric disease or treatment (Table S1). All subjects deceased suddenly without prolonged agonal phase and had clear neuropathology and toxicology exams. Diagnostic and Statistical Manual of Mental Disorders (DSM) IV diagnosis was determined by validated psychological autopsy using the Structured Clinical Interview for DSM (SCID) I Kelly and Mann, 1996). Recent (last 3 months) life event-related stress was quantified using the St. Paul-Ramsey Life Experience Scale. Given the role of the ventral (anterior in humans) hippocampus in emotion (Fudge et al., 2012), we examined *KLF9* expression in the anterior hippocampal DG (Figures 7A and 7B, red outline). Remarkably, we found that women, but not men, with MDD showed increased levels of *KLF9* expression in anterior DG compared with controls (Figure 7C). In addition, a significant positive correlation between anterior DG *KLF9* expression and the severity of recent stressful life events was found in women but not men (Figure 7D). Our results from this preliminary sample suggest that stressful experiences may upregulate *KLF9* expression in the DG in a gender-specific manner and possibly be linked to the pathogenesis of MDD and/or suicide.

## DISCUSSION

Understanding how chronic stress engenders maladaptive fear responses will edify treatments for stress-related psychopathologies such as PTSD and MDD. Essential to this challenge is instantiation of the transcriptional and non-genomic mechanisms that link physiological effectors of chronic stress with changes in dendritic spines in brain regions such as the hippocampus. Our studies identify a role for Klf9 as one such mediator whose expression is regulated by stress in both the rodent and human hippocampus. Importantly, downregulation of *Klf9* in excitatory forebrain neurons prevents chronic stress-induced enlargement of dendritic spines in the ventral hippocampus and dysregulation of fear.

Our studies show that acute restraint stress, as well as acute CORT treatment (Carter et al., 2013), elevates *Klf9* expression in the hippocampus. In contrast, CRS does not maintain the elevation in *Klf9* expression in principal cell populations of the hippocampus induced by the acute phase of stressor. The restoration of *Klf9* expression levels to baseline following the acute phase of stressor may be mediated by glucocorticoid receptor (GR) signaling (Bagamasbad et al., 2012; Bonett et al., 2009; Datson et al., 2011) or other physiological regulators of *Klf9* expression such as neural activity (Flavell et al., 2008; Lin et al., 2008; Scobie et al., 2009), thyroid hormone (Denver et al., 1999), and progesterone and estrogen (Knoedler and Denver, 2014). To determine whether the transient *Klf9* upregulation seen following onset of stressor is adaptive or maladaptive, we generated mice in which we could inducibly silence *Klf9* expression in excitatory forebrain neurons. Inducible *Klf9* silencing in stress-naive mice did not affect contextual fear acquisition or generalization. However, *Klf9* silencing prior to and during chronic CORT treatment or CRS prevented fear generalization in a neutral context or potentiation of acquisition of contextual fear in male and female mice. Importantly, silencing *Klf9* following the onset of the stressor did not prevent the deleterious effects of stress on contextual fear generalization and acquisition in male and female mice, respectively. These data suggest that elevation of *Klf9* levels in response to acute stress may represent a potential pathogenic mechanism that produces lasting mal-adaptations altering contextual fear memory processing. In this regard, it is noteworthy that *Klf9* has a Sin3A interaction domain and can recruit the mSin3A-corepressor complex associated with histone deacetylase enzymatic activity (Zhang et al., 2001; Moore et al., 2011). We hypothesize that transient upregulation of *Klf9* expression by acute stress promotes long-lasting stress-induced mal-adaptations through chromatin remodeling.

Because of the well-characterized role of the hippocampus in contextual fear memory processing (Fanselow and Dong, 2010; Maren et al., 2013; Strange et al., 2014; Tonegawa et al., 2015), we analyzed dendritic spines in DG and CA1 subregions of dorsal and ventral hippocampus. Our studies identify vDG and ventral CA1 (vCA1) as two putative loci of vulnerability to chronic CORT and CRS in male and female mice, respectively. Both of these subregions exhibited chronic stress-induced enlargement of dendritic spines, and silencing of *Klf9* expression prevented these alterations. In addition, we found that CRS induced opposing effects on spine width in the vCA1 of male (decreased spine width) and female (increased spine width) mice. This observation resonates with a previous study showing how the same stressful experience engenders opposing structural changes in hippocampus of male and female mice (Shors et al., 2001).

How might enlarged dendritic spines in these regions promote maladaptive fear responses? One hypothesis is that CRS-induced enlarged spines are inefficient (A.B. and A.S., unpublished data) and disruption of encoding in ventral DG (vDG) and vCA1 (Bagot et al., 2015) impairs hippocampal dependent regulation of amygdala, prefrontal cortex, and nucleus accumbens (Ciocchi et al., 2015; Guo et al., 2018; Xu et al., 2016) to gate stress-dependent aberrations in fear responses.

Klf9’s primary role is to constrain neuronal connectivity, as exemplified in studies showing that acute overexpression of *Klf9* negatively regulates dendritic spine number (McAvoy et al., 2016) and neurite growth (Moore et al., 2009, 2011). How then do these functions of *Klf9* explain the reversal in stress-induced dendritic spine enlargement following *Klf9* silencing? Our data lead us to propose that *Klf9* upregulation exerts different biological effects on dendritic spines in stress-naive mice and under conditions of stress exposure, a hypothesis that remains to be tested. Klf9 is both an activator and a repressor of transcription (Moore et al., 2011) and may be regulated in different ways in naive mice and under conditions of stress exposure. It is plausible that under conditions of stress exposure, but not in stress-naive mice, *Klf9* silencing suppresses availability of factors that are recruited by chronic stress for spine enlargement. These factors could be cytoskeletal molecules, cell-surface receptors, or components of endocytic machinery that facilitate dendritic spine growth.

Our human postmortem hippocampal tissue analysis uncovered elevated levels of *KLF9* mRNA expression in women but not men with MDD-suicide and a positive correlation with severity of recent stressful life events in women but not men. These data suggest that elevation in *KLF9* levels may represent a potential pathogenic mechanism of MDD and/or suicide. By integrating neural activity, sex, and stress hormones, Klf9 may permit stress-induced adaptations in a sex-specific manner. Upregulation of *KLF9* expression levels may render threat-processing circuits inefficient in calibrating fear responses. Further studies are needed to determine if genetic mutations in the human population that impair *KLF9* downregulation in response to stress confer vulnerability to MDD and PTSD.

## EXPERIMENTAL PROCEDURES

### Animal Studies

#### Animal Care

Male and female mice were housed four per cage in a 12 hr (7 a.m. to 7 p.m.) light/dark colony room at 22°C–24°C with *ad libitum* access to food and water. Behavioral experiments took place between 8 a.m. and 6 p.m. All animals were handled and experiments were conducted in accordance with procedures approved by the Institutional Animal Care and Use Committee at the Massachusetts General Hospital and Boston University in accordance with NIH guidelines.

#### Mouse Lines

Inducible and reversible silencing of Klf9 in forebrain excitatory neurons was achieved by generating a bigenic tTS:Tre-*Klf9* mouse line resulting from breeding Tre-*Klf9* mouse line (McAvoy et al., 2016) with CamKIIα-tTS line (Richardson-Jones et al., 2011). This mouse line was homozygous for the tetO insertion (Tre-*Klf9*/Tre-*Klf9*), had one copy of CamKIIα-tTS, and was fed DOX ad libitum throughout life to allow the expression of endogenous Klf9 in forebrain excitatory neurons.

Spine analyses was carried out by generating a trigenic mouse line resulting from breeding tTS:Tre-*Klf9* mouse line with Thy1-GFP (M Line) mice (Feng et al., 2000), which were purchased from The Jackson Laboratory (strain 007788) and were maintained by crossing with homozygous Tre-*Klf9*. Tail DNA from all offspring was genotyped by PCR to detect the presence of each transgene separately. All experiments were conducted with 8- to 17-week-old mice (unless indicated otherwise).

#### Drugs

CORT was prepared as previously described (David et al., 2009). Specifically, 35 μg/mL (equivalent to 5 mg/kg/day) CORT (27840; Sigma-Aldrich, St. Louis, MO, USA) was dissolved in 0.45% 2-hydroxypropyl cyclodextrin (332593; Sigma-Aldrich) and delivered in lightproof bottles, available *ad libitum* in drinking water. Control mice received 0.45% β-cyclodextrin in drinking water (C4767; Sigma-Aldrich). DOX was provided in chow containing 200 mg/kg DOX (S3888; Bioserv, Flemington, NJ, USA) fed *ad libitum* throughout life. Control chow (S4207; Bioserv) was used when DOX was withdrawn from the diet.

#### Acute and CRS

Acute restraint stress consisted in 6 hr of complete immobilization using disposable mouse DecapiCone restrainers (DC M200; Braintree Scientific, Braintree, MA, USA). CRS consisted of 6 hrs of complete immobilization using disposable mouse DecapiCone restrainers each day for 14 consecutive days before behavioral testing.

#### In Situ *Hybridization*

*In situ* hybridization (ISH) was performed using *Klf9*-specific riboprobes as previously described (McAvoy et al., 2016) using dioxygenin-labeled riboprobes on 35 μm cryosections generated from perfused tissue. Premixed RNA labeling nucleotide mixes containing digoxigenin-labeled UTP (Roche Molecular Biochemicals) were used to generate RNA riboprobes. Riboprobes were purified on G-50 Microspin columns (GE Healthcare). Probe concentration was confirmed by NanoDrop prior to the addition of formamide. Briefly, sections were mounted on charged glass (Superfrost Plus) slides and postfixed for in 4% paraformaldehyde (PFA). Sections were then washed in DEPC-treated PBS, treated with Proteinase K (40 μg/mL final), washed again in DEPC-treated PBS, and then acetylated. Following prehybridization, sections were incubated with riboprobe overnight at 58° and washed in decreasing concentrations of saline sodium citrate (SSC) buffer, and immunological detection was carried out with anti-dioxygenin antibody conjugated with alkaline phosphatase (Roche). Color reaction was carried out with nitro blue tetrazolium (NBT)/5-bromo-4-chloro-3-indolyl-phosphate (BCIP). Color reaction times were identical for both treatment groups. For quantification, two to four color images per region per mouse were analyzed using the mean intensity function in ImageJ (NIH). All images were captured using the same light intensity and exposure times. The mean intensity of the region of interest (minus mean intensity of a selected background region) was averaged across images for each mouse and each treatment group.

#### CFC Discrimination Learning

The conditioning chambers (18 × 18 × 30 cm) consisted of two clear Plexiglas walls and ceiling, two metal walls, and a stainless steel grid floor (Coulbourn Instruments, Whitehall, PA, USA). The conditioning chambers were placed inside a ventilated, sound-dampening isolation cubicles and lit by house lights mounted on one wall (Coulbourn Instruments). FreezeFrame and FreezeView software (Actimetrics, Wilmette, IL, USA) was used for recording and analyzing freezing behavior, respectively. For the training context (designated A throughout), the cubicle door was closed, the fan and house light were on, a light cue was on, stainless-steel bars were exposed, silver wall panels were used, and each conditioning chamber was cleaned with 70% ethanol between each trial. Context B was a modified version of A by covering the stainless-steel bars with a solid floor covered with bedding, black wall panels were used (covering 30% of total wall surface), 15 cm high curved green plastic inserts covered the bottom half of the walls, and the house fan and lights were turned off. The cubicle door was left ajar, and white noise was delivered through built-in speakers for the duration of the testing. The bedding was changed between trials. Context C consisted of a disposable 2.4 L white paper bucket placed out of the cubicle in the same experimental room as contexts A and B.

The CFC protocol consisted in a single 2 s footshock of 0.7 mA which was delivered 180 s after placement of the mouse in the training context A. The mouse was taken out 20 s after termination of the footshock. This procedure was repeated for 3 days (24 hr apart). On day 4, 50% of the animals were first tested in context A or B in the morning and context B or A in the afternoon. In some instances, animals were also tested in context C, which took place after both exposures to contexts A and B. No footshocks were delivered during the test sessions. Mice were allowed to rest for 1–2 hr between tests. Freezing behavior over the initial 180 s was used to assess discrimination between both contexts. The discrimination ratio was calculated as (freezing in training context — freezing in similar context)/(freezing in training context + freezing in similar context).

#### Immunohistochemistry

Mice were anesthetized with ketamine and xylazine (100 mg/kg and 15 mg/kg, intraperitoneal [i.p.]; Patterson Veterinary Supply, Devens, MA, USA) and transcardially perfused with DEPC-treated PBS (10 mM PBS [pH 7.5]) at 4°C, followed by 4% PFA in DEPC-treated PBS at 4°C. Brains were postfixed overnight in the same solution at 4°C, then cryoprotected in DEPC-treated PBS sucrose (30% w/v) and stored at 4°C before freezing in optimal cutting temperature (OCT) on dry ice. Coronal serial sections (35 μm) were obtained using a Leica cryostat in six matched sets. Sections were stored in PBS with 0.01% sodium azide at 4°C. On day 1, free-floating sections were rinsed three times for 10 min in PBS, followed by a permeabilization step 15 min in 0.2% Triton X-100 in PBS. The sections were rinsed another three times for 10 min in PBS and 2 hr with a blocking buffer (10% w/v natural donkey serum [NDS]). After three rinses in PBS, incubation with primary antibodies rabbit anti-GFP (Life Technologies A11122, 1:500 [Antibodyregistry.org: AB_221569]) was carried out with shaking at 4°C overnight. On day 2, sections were rinsed three times for 10 min in PBS and incubated for 90 min with a donkey anti-rabbit fluorescein isothiocyanate (FITC)-coupled secondary antibody (1:500; Jackson ImmunoResearch). Sections were rinsed three times for 10 min in PBS before mounting in PBS and coverslipped with Fluoromount.

#### Image Analysis of Dendritic Spines

Quantification of dendritic spines was conducted as previously described (McAvoy et al., 2016). Specifically, confocal z stack images were acquired using a Nikon A1R Si confocal laser, a TiE inverted research microscope, and NIS Elements software. Imaging was performed using a 60× objective, plus 1.5× optical zoom and 6× digital zoom. For spine imaging, confocal 2.1 μM z stacks (2,048 resolution) with 0.3 μM step size were taken centered on dendritic segment. Z stacks were flattened using the maximum intensity projection, and flattened images were quantified using ImageJ. For spine density, spines were counted manually for at least 80 μM of dendritic length per region per mouse. The Edge fitter plugin (www.ghoshlab.org) was used to measure head diameter (at the widest point of the spine head), while length was measured manually from dendrite to the furthest point of the spine head. More than 150 spines were analyzed per region per mouse to calculate spine size distribution. In DG, images were obtained in the outer molecular layer (defined as the third of the molecular layer furthest from the granule cell layer). In CA1, images were obtained in the stratum radiatum, which was defined as the two-thirds of the dendritic tree ventral to the pyramidal layer. All imaging and quantification were performed by an investigator blind to treatment.

### Human Studies

#### Brain Collection

Brain tissue was obtained from the Brain Collection of the New York State Psychiatric Institute at Columbia University, which includes brain samples from the Republic of Macedonia. Brain tissue collection was conducted with institutional review board (IRB) approval and consent obtained from all informants.

#### Subject Selection and Matching Procedure

All subjects are deceased. Our IRB has determined that this postmortem work is not human subjects research. The only involvement of live individuals is as informants for psychological autopsy interviews. Subjects were diagnosed using our validated psychological autopsy for DSM axis I and II diagnoses (Kelly and Mann, 1996). History of lifetime psychiatric disorders and recent medication records were obtained. Clinical evaluation included global functioning as measured using the Global Assessment Scale (GAS; score range 0–100, with 1–10 indicating danger of harming self or others, inability to maintain personal hygiene, or serious suicide attempt, and 91–100 indicating no problems, superior functioning in several areas, or admired by others because of positive qualities) (Endicott et al., 1976), as per DSM IV axis V; recent (over the past 2 month period) life event-associated stress (related to primary support group, social environment, education, occupation, housing, finances, health, legal, or other psychosocial and environmental factors), quantified using St. Paul-Ramsey Life Experience Scale (SPRS) total score and DSM-IV axis IV severity (score range 1–7: none, minimal, mild, moderate, severe, extreme, and catastrophic) (Roy et al., 1986); documentation of age at MDD onset; and number of major depressive episodes (MDEs) lifetime.

We studied 24 subjects (Table S1): age- and sex-matched individuals with no axis I or axis II cluster B personality disorder and death not by suicide (controls) and MDD cases (n = 12 each), with six women and men in each group. All subjects died by sudden death, and we limited postmortem interval (PMI) to 24 hr. We screened brain tissue for low pH, excluding tissue that had been damaged and showed low pH, and we controlled for PMI because it affects brain proteins and mRNA (Lewis, 2002; Li et al., 2003).

Exclusion criteria were positive toxicology for psychoactive drugs or alcohol, alcoholism-associated liver changes, suicide attempt history, intellectual disability, AIDS, chronic illness, positive neuropathology, undetermined death, resuscitation with prolonged (>10 min) hypoxia, received prescription of psychotropic medications in the 3 months prior to death, and long agonal states and chronic diseases. The presence of diseases affecting the brain was excluded using clinical data, psychological autopsy interviews, and neuropathological exams. Neuropathology excluded microvascular disease, including white matter lacunae.

#### Brain Tissue Processing

At brain collection, 2-cm-thick coronal blocks of the right hemisphere were flash-frozen in liquid freon (−20°C) and stored at −80°C. Tissue samples were fixed in formalin for neuropathological examination. Brain pH determination (Harrison et al., 1995) and toxicology were performed on cerebellar samples and blood. More than 30 drugs were screened for and quantified, including amphetamine, cocaine, fluoxetine, sertraline, paroxetine, fluvoxamine, amitriptyline, nortriptyline, imipramine, citalopram, chlorimipramine, diazepam, alprazolam, buspirone, methadone, olanzapine, clozapine, and haloperidol.

The hippocampal formation was dissected from frozen coronal blocks, fixed in 4% PFA at 4°C, cryoprotected in 30% sucrose, sectioned at 50 μm on a sliding microtome (Microm HM440E), and stored in 40-well boxes at −20°C in cryoprotectant (30% ethylene glycol in 0.1 M PBS). One section every 500 μm was set aside for Nissl staining during the sectioning procedure. Nissl-stained sections were later used for anatomical alignment along the DG rostro-caudal axis of sections processed for ISH.

#### ISH in Human Tissue

In order to quantify the concentration of *KLF9* mRNA in the anterior human DG, we performed an ISH assay on three anterior hippocampus sections, using an ^35^S-radiolabeled probe (200 bp: Nt 185-395 *Homo sapiens Klf9*, mRNA [cDNA clone MGC:97030 IMAGE:7262239], complete cds Sequence ID: BC069431.1), directed to *KLF9* mRNA. The probe was labeled using 10 μCi/mL of cytidine 5′ (α-thio) [^35^S] (PerkinElmer) and T3 polymerase and then incubated at 37°C for 90 min. Next, we added RQ1 RNase-Free DNase and incubated at 37°C for 30 min. Immediately after digesting the DNA template, the RNA probe was purified using RNeasy Kit (QIAGEN, Hilden, Germany), as directed by the manufacturer.

To prepare the brain tissue for hybridization with the probe described above, 50-μm-thick coronal sections of PFA-fixed hippocampus were washed in 0.05 M phosphate (pH 7.4) five times, 20 min each, in an orbital shaker at 60 rpm. The brain sections were then mounted on gelatin-coated microscope slide glasses and desiccated for 3 hr. The tissue was then hydrated in 1:10 diluted Dulbecco’s PBS for 5 min and then incubated in a solution of 0.15 M sodium chloride, 0.1 M triethanolamine, 50 mM hydrochloric acid, and 25 mM acetic anhydride for 10 min. Then, the tissue was incubated in 2 × SDS (0.3 M sodium chloride, 30 mM sodium citrate) for 1 min, followed by increasing concentrations of ethanol, followed by 10 min in chloroform and 1 min in 100% ethanol and then 95% ethanol.

To hybridize the probe with the tissue, the slides were dried for 1 min and then hybridized with 100 μL of hybridization solution containing 1 × 10^6^ dpm of the *KLF9* probe per slide, covered with a coverslip and placed in humidifying chamber, at 55°C for 18 hr. The probe was then washed off the tissue using the following procedure: (1) 4 × SSC containing 0.07 M 2-mercaptoethanol for 15 min; (2) 4 × SSC for 15 min; (3) (1:1) formamide: buffer (0.6 M sodium chloride, 0.04 M Tris base, 0.01 mM hydrochloric acid, and 1 mM EDTA) for 20 min at 55°C; the probe was then digested in 10 μg/mL RNase A for 30 min at 37°C; (4) serial washes of decreasing concentrations from 2 × to 0.5 × SSC; (5) 0.1 × SSC for 30 min at 55°C; and (6) 0.1 × SSC at room temperature and last in 60% ethanol and 0.33% ammonium acetate.

Then, glass slides with the mounted hybridized tissue were dried and exposed in the dark to a BioMax MR film (Carestream Kodak) for 10 days, accompanied by standards of radioactivity, ranging from 0 to 2.15 nCi/mg (^14^C catalog number ARC 146A; American Radiolabeled Chemicals).

The film was then developed using developer and fixer solutions (Carestream Kodak), and the autoradiographs of the tissue and the standards were digitized using an Epson Expression 11000XL scanner at 3,200 dots per inch (dpi).

#### Densitometry Analysis of KLF9 mRNA Content in DG

The concentration of *KLF9* mRNA was quantified using Densita software (MBF Bioscience, Willinston, VT, USA), as explained below. Densita software calculated the relative optical density (ROD) of the scanned autoradiograms of radioactivity standards and created a graph of the concentration of each standard, in nanocuries per milligram, versus their relative optical density. We calculated the relative optical density produced by the hybridization of the *KLF9* ^35^S probe in hippocampal DG in digitized autoradiographs. We first contoured the DG area using sections that had been stained for Nissl as anatomical reference. Contours were then superimposed on the digitized ISH autoradiographs, and the relative optical density of each DG contour was determined using Densita. The relative optical densities were entered into the standard curve to obtain the correspondent radioactivity concentration. Densita software corrected the *KLF9* concentrations using the specific activity of the radioisotope in the probe and the rate decay of the radioisotope. Finally, the concentration of mRNA *KLF9* in the contour of DG areas was calculated on three sections of the anterior portion of the hippocampus as average concentration in nanocuries per milligram of tissue. The final DG relative optical density was background-corrected, subtracting the background signal in the tissue white matter and on the slide (outside the section) from the relative optical density of the DG in the same section.

### Data Availability

All relevant data are available from the authors.

### Statistical Analysis

All analyses were performed by an investigator blinded to treatment and/or genotype using SPSS version 24 for Mac and GraphPad Prism version 7. ISH data (mean ± SEM) were analyzed using unpaired two-tailed Student’s t test or one-way ANOVA followed by Tukey’s multiple-comparisons post hoc test. p values were corrected for multiple comparison to q values to control for false discovery rate (FDR) (desired FDR [Q] = 5%) (Benjamini et al., 2006). Behavioral and morphological data were analyzed using mixed-factor two-way ANOVA (with or without repeated measures over time) followed by Tukey’s multiple-comparisons test when appropriate (only if interaction p < 0.05). Linear regression was used to test correlations between recent stressful life event severity score and *KLF9* mRNA density in human tissue. Univariate ANOVA was used to test *KLF9* mRNA density differences in controls and MDD, men and women, using age, St. Paul-Ramsey Life Experience Scale score, PMI, pH, and age as covariates. In any case, significance was set at p < 0.05.

## Supplementary Material

Supp Table 1

2

## Highlights

- Chronic stress (CS) transiently elevates *Klf9* expression in the hippocampus of mice
- *Klf9* silencing in male and female mice prevents CS-induced spine alterations
- *Klf9* silencing in male and female mice prevents CS-induced maladaptive fear responses
- Hippocampal *KLF9* levels are increased in women with CS exposure and MDD
