# Supplementary material for: Targeting Kruppel-like Factor 9 in Excitatory Neurons Protects against Chronic Stress-Induced Impairments in Dendritic Spines and Fear Responses
Source: Cell Rep. Author manuscript; Available in PMC 2020 Aug 28. (PMC7453932; doi:10.1016/j.celrep.2018.05.040)
Supplement: 2 [file NIHMS1616560-supplement-2.pdf]

Cell Reports, Volume 23

Supplemental Information

Targeting Kruppel-like Factor 9 in Excitatory Neurons Protects against Chronic Stress-Induced Impairments in Dendritic Spines and Fear Responses

Antoine Besnard, Tomer Langberg, Sally Levinson, Duong Chu, Cinzia Vicidomini, Kimberly N. Scobie, Andrew J. Dwork, Victoria Arango, Gorazd B. Rosoklija, J. John Mann, René Hen, E. David Leonardo, Maura Boldrini, and Amar Sahay

Figure S1

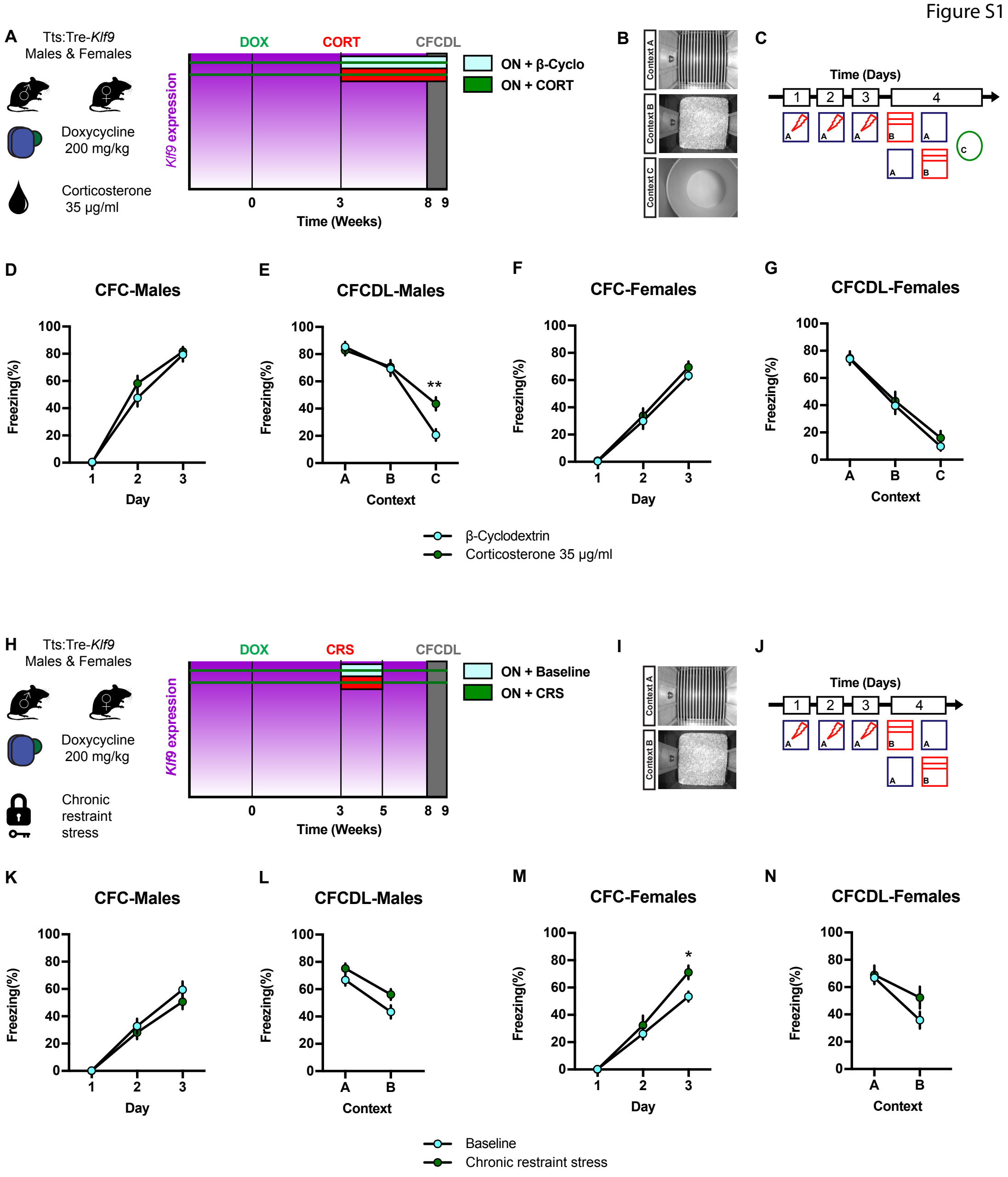
A
Tts:Tre-Klf9
Males & Females
Doxycycline
200 mg/kg
Corticosterone
35 µg/ml
DOX
CORT
CFCDL
Klf9 expression
ON + β-Cyclo
ON + CORT
Time (Weeks)
B
Context A
Context B
Context C
C
Time (Days)
D
CFC-Males
E
CFCDL-Males
F
CFC-Females
G
CFCDL-Females
Freezing(%)
Day
Context
β-Cyclodextrin
Corticosterone 35 µg/ml
H
Tts:Tre-Klf9
Males & Females
Doxycycline
200 mg/kg
Chronic
restraint
stress
DOX
CRS
CFCDL
ON + Baseline
ON + CRS
Time (Weeks)
I
J
Time (Days)
K
CFC-Males
L
CFCDL-Males
M
CFC-Females
N
CFCDL-Females
Baseline
Chronic restraint stress

Figure S1. Differential effects of chronic corticosterone and chronic restraint stress on contextual fear conditioning and generalization in male and female mice, Related to Figures 3 and 5

A) Schematic representation of tTS:Tre-*Klf9* male and female mice raised with doxycycline and exposed to corticosterone or β-cyclodextrin control. **B)** CFCDL procedure in which mice were trained to discriminate between a footshock delivered in context A and safe contexts B and C. **C)** Schematic representation of CFCDL timeline consisting of 3 days of training in context A and a discrimination tests on day 4. **D)** Freezing behavior of male mice on days 1-3. Data (means ± SEM; n= 11,12 mice per group) were analyzed using mixed factor two-way ANOVA (repeated measure over time): time $F_{(2,42)} = 253.6$, $P < 0.001$; corticosterone $F_{(1,21)} = 0.88$, NS; interaction $F_{(2,42)} = 1.3$, NS. **E)** Freezing behavior of male mice on day 4. Data (means ± SEM; n= 11,12 mice per group) were analyzed using mixed factor two-way ANOVA (repeated measure over time): context $F_{(2,42)} = 166.8$, $P < 0.001$; corticosterone $F_{(1,21)} = 1.76$, NS; interaction $F_{(2,42)} = 10.74$, $P < 0.001$, **$p < 0.01$, corticosterone versus β-cyclodextrin. **F)** Freezing behavior of female mice on days 1-3. Data (means ± SEM; n= 10,11 mice per group) were analyzed using mixed factor two-way ANOVA (repeated measure over time): time $F_{(2,38)} = 209.5$, $P < 0.001$; corticosterone $F_{(1,19)} = 0.77$, NS; interaction $F_{(2,38)} = 0.4$, NS. **G)** Freezing behavior of female mice on day 4. Data (means ± SEM; n= 10,11 mice per group) were analyzed using mixed factor two-way ANOVA (repeated measure over time): context $F_{(2,38)} = 174.6$, $P < 0.001$; corticosterone $F_{(1,19)} = 0.29$, NS; interaction $F_{(2,38)} = 0.36$, NS. **H)** Schematic representation of tTS:Tre-*Klf9* male and female mice raised with doxycycline and CRS timeline . **I)** CFCDL procedure in which mice were trained to discriminate between a footshock delivered in context A and safe context B. **J)** Schematic representation of CFCDL timeline consisting of 3 days of training in context A and a discrimination tests on day 4. **K)** Freezing behavior of male mice on days 1-3. Data (means ± SEM; n= 12,12 mice per group) were analyzed using mixed factor two-way ANOVA (repeated measure over time): time $F_{(2,44)} = 96.92$, $P < 0.001$; CRS $F_{(1,22)} = 1.04$, NS; interaction $F_{(2,44)} = 0.61$, NS. **L)** Freezing behavior of male mice on day 4. Data (means ± SEM; n= 12,12 mice per group) were analyzed using mixed factor two-way ANOVA (repeated measure over time): context $F_{(1,22)} = 36.93$, $P < 0.001$; CRS $F_{(1,22)} = 4.89$, $P < 0.05$; interaction $F_{(1,22)} = 0.36$, NS. **M)** Freezing behavior of female mice on days 1-3. Data (means ± SEM; n= 12,10 mice per group) were analyzed using mixed factor two-way ANOVA (repeated measure over time): time $F_{(2,40)} = 158.9$, $P < 0.001$; CRS $F_{(1,20)} = 3.54$, NS; interaction $F_{(2,40)} = 3.4$, $P < 0.05$, *$p < 0.05$, CRS versus baseline. **N)** Freezing behavior of female mice on day 4. Data (means ± SEM; n= 12,10 mice per group) were analyzed using mixed factor two-way ANOVA (repeated measure over time): context $F_{(1,20)} = 25.2$, $P < 0.001$; CRS $F_{(1,20)} = 1.53$, NS; interaction $F_{(1,20)} = 2.32$, NS.

Figure S2

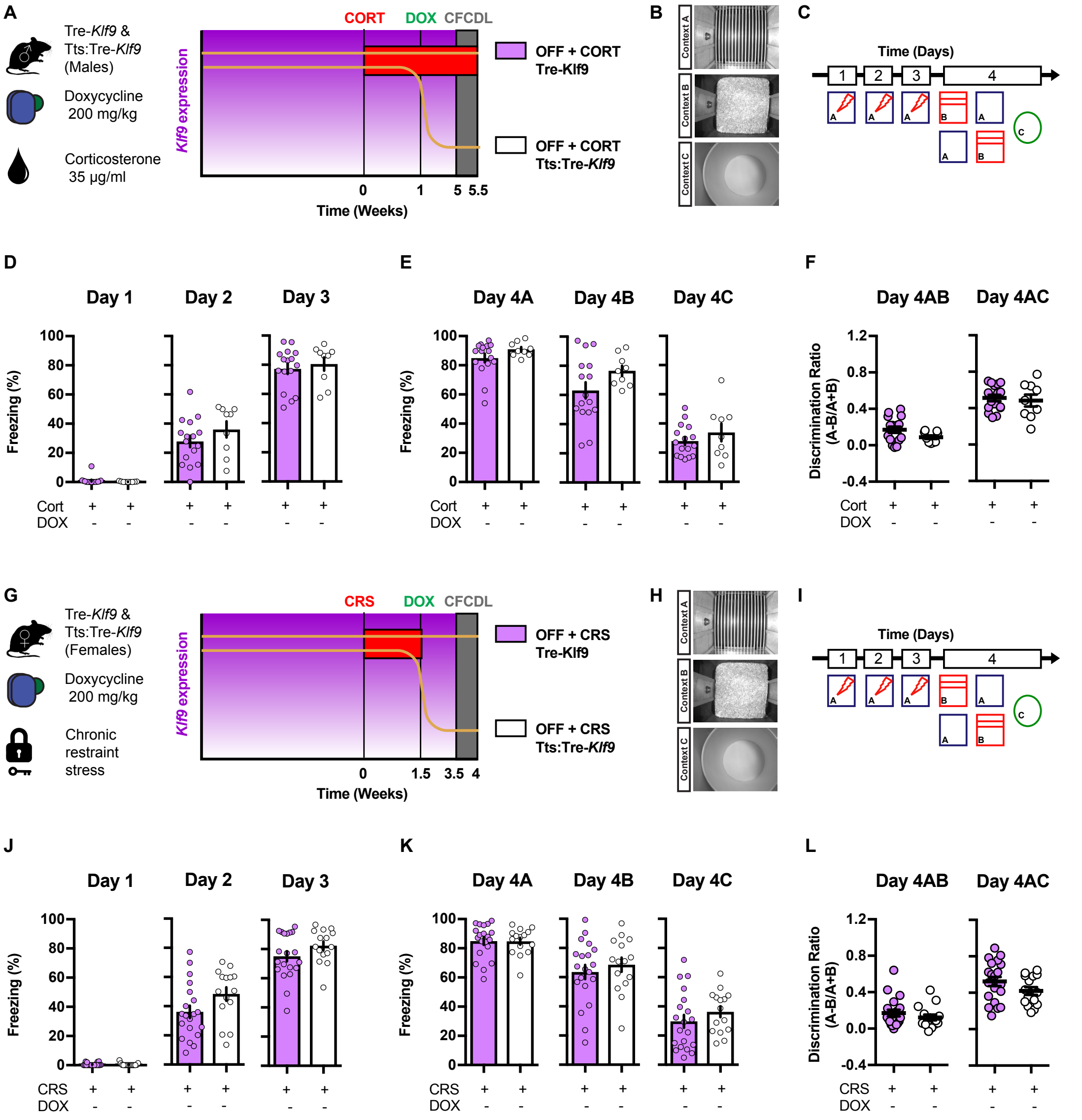

A
Tre-Klf9 &
Tts:Tre-Klf9
(Males)
Doxycycline
200 mg/kg
Corticosterone
35 µg/ml
CORT
DOX
CFCDL
Klf9 expression
0
1
5
5.5
Time (Weeks)
OFF + CORT
Tre-Klf9
OFF + CORT
Tts:Tre-Klf9
B
Context A
Context B
Context C
C
Time (Days)
1
2
3
4
D
Day 1
Day 2
Day 3
Freezing (%)
Cort
DOX
E
Day 4A
Day 4B
Day 4C
F
Day 4AB
Day 4AC
Discrimination Ratio
(A-B/A+B)
G
Tre-Klf9 &
Tts:Tre-Klf9
(Females)
Doxycycline
200 mg/kg
Chronic
restraint
stress
CRS
DOX
CFCDL
0
1.5
3.5
4
Time (Weeks)
OFF + CRS
Tre-Klf9
OFF + CRS
Tts:Tre-Klf9
H
I
J
K
L
CRS
DOX

Figure S2. Inducible silencing of *Klf9* expression in forebrain excitatory neurons of male and female mice following onset of chronic corticosterone treatment and chronic restraint stress exposure does not prevent stress-induced adaptations in contextual fear, Related to Figures 3 and 5

A) Schematic representation of doxycycline and corticosterone diet schedules for Tre-*Klf9* and tTS:Tre-*Klf9* male mice. **B)** CFCDL procedure in which mice were trained to discriminate between a footshock delivered in context A and safe contexts B and C. **C)** Schematic representation of CFCDL timeline consisting of 3 days of training in context A and discrimination tests on day 4. **D)** Freezing behavior on day 1, 2 and 3. Data (means ± SEM; n= 16,9 mice per group) were analyzed using unpaired two-tailed Student's T-test (NS). **E)** Freezing behavior on day 4 in context A, B and C. Data (means ± SEM; n= 16,9 mice per group) were analyzed using unpaired two-tailed Student's T-test (NS). **F)** Discrimination ratio calculated for contexts A, B and C. Data (means ± SEM; n= 16,9 mice per group) were analyzed using unpaired two-tailed Student's T-test (NS). **G)** Schematic representation of doxycycline and chronic restraint stress schedules for Tre-*Klf9* and tTS:Tre-*Klf9* female mice. **H)** CFCDL procedure in which mice were trained to discriminate between a footshock delivered in context A and safe contexts B and C. **I)** Schematic representation of CFCDL timeline showing 3 days of training in context A and discrimination tests on day 4. **J)** Freezing behavior on day 1, 2 and 3. Data (means ± SEM; n= 20,15 mice per group) were analyzed using unpaired two-tailed Student's T-test (NS). **K)** Freezing behavior on day 4 in context A, B and C. Data (means ± SEM; n= 20,15 mice per group) were analyzed using unpaired two-tailed Student's T-test (NS). **L)** Discrimination ratio calculated for contexts A, B and C. Data (means ± SEM; n= 20,15 mice per group) were analyzed using unpaired two-tailed Student's T-test (NS).

Figure S3

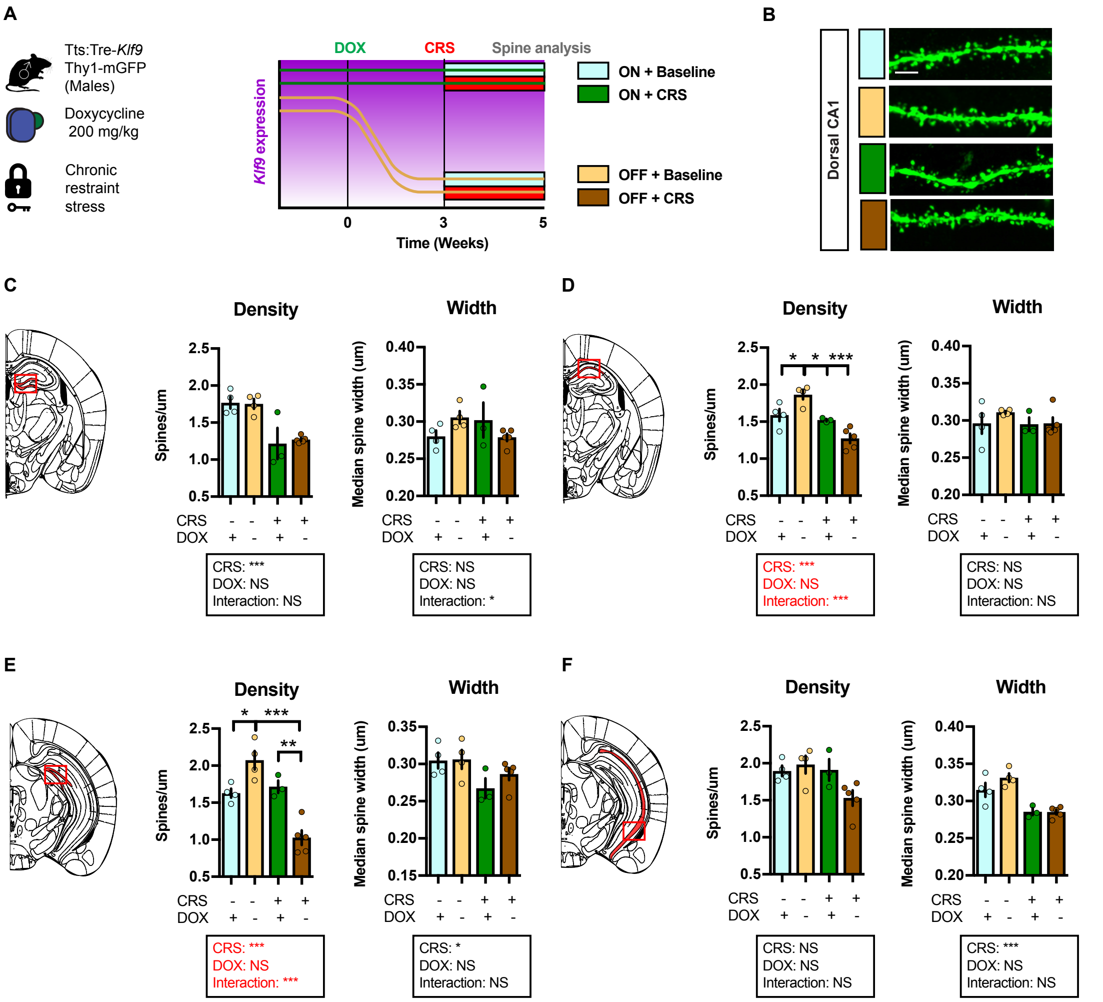

A
Tts:Tre-Klf9
Thy1-mGFP
(Males)
Doxycycline
200 mg/kg
Chronic
restraint
stress
DOX
CRS
Spine analysis
Klf9 expression
Time (Weeks)
ON + Baseline
ON + CRS
OFF + Baseline
OFF + CRS
B
Dorsal CA1
C
Density
Width
Spines/um
Median spine width (um)
CRS: ***
DOX: NS
Interaction: NS
CRS: NS
DOX: NS
Interaction: *
D
CRS: ***
DOX: NS
Interaction: ***
CRS: NS
DOX: NS
Interaction: NS
E
CRS: ***
DOX: NS
Interaction: ***
CRS: *
DOX: NS
Interaction: NS
F
CRS: NS
DOX: NS
Interaction: NS
CRS: ***
DOX: NS
Interaction: NS

Figure S3. Inducible silencing of *Klf9* expression in forebrain excitatory neurons of male mice does not prevent CRS decrease in dendritic spines in ventral CA1, Related to Figure 6

A) Schematic representation of tTS:Tre-*Klf9*:Thy1-mGFP/+ male mice with different doxycycline diet schedules and chronic restraint stress timeline. **B)** Maximum intensity projection confocal images of individual dendritic segments from the stratum radiatum in dorsal CA1. Representative images for 3-5 independent animals per group. Scale bar: 2 µm. **C)** Spine density and width in dorsal DG (red box). Data (means ± SEM; n= 4,4,3,5 mice per group) were analyzed using mixed factor two-way ANOVA: Density: CRS $F_{(1,12)} = 30.47$, $P < 0.001$; doxycycline $F_{(1, 12)} = 0.03$, NS; interaction $F_{(1,12)} = 0.13$, NS; Width: CRS $F_{(1,12)} = 0.05$, NS; doxycycline $F_{(1, 12)} = 0.01$, NS; interaction $F_{(1,12)} = 5.12$, $P < 0.05$. **D)** Spine density and width in dorsal CA1 (red box). Data (means ± SEM; n= 4,4,3,5 mice per group) were analyzed using mixed factor two-way ANOVA: Density: CRS $F_{(1,12)} = 23.47$, $P < 0.001$; doxycycline $F_{(1, 12)} = 0.04$, NS; interaction $F_{(1,12)} = 14.93$, $P < 0.01$, *$p < 0.05$, ***$p < 0.001$; Width: CRS $F_{(1,12)} = 0.72$, NS; doxycycline $F_{(1, 12)} = 0.72$, NS; interaction $F_{(1,12)} = 0.53$, NS. **E)** Spine density and width in ventral DG (red box). Data (means ± SEM; n= 4,4,3,5 mice per group) were analyzed using mixed factor two-way ANOVA: Density: CRS $F_{(1,12)} = 22.39$, $P < 0.001$; doxycycline $F_{(1, 12)} = 1.49$, NS; interaction $F_{(1,12)} = 31.36$, $P < 0.001$, *$p < 0.05$, **$p < 0.001$, ***$p < 0.001$; Width: CRS $F_{(1,12)} = 6.68$, *$p < 0.05$; doxycycline $F_{(1, 12)} = 0.87$, NS; interaction $F_{(1,12)} = 0.63$, NS. **F)** Spine density and width in ventral CA1 (red box). Data (means ± SEM; n= 4,4,3,5 mice per group) were analyzed using mixed factor two-way ANOVA: Density: CRS $F_{(1,12)} = 3.84$, NS; doxycycline $F_{(1, 12)} = 1.76$, NS; interaction $F_{(1,12)} = 4.38$, NS; Width: CRS $F_{(1,12)} = 37.14$, ***$p < 0.001$; doxycycline $F_{(1, 12)} = 1.67$, NS; interaction $F_{(1,12)} = 1.8$, NS.
